# *Spissistilus festinus* (Hemiptera: Membracidae) susceptibility to six generalist predators

**DOI:** 10.1371/journal.pone.0242775

**Published:** 2020-11-30

**Authors:** Cindy R. Kron, Mark S. Sisterson

**Affiliations:** 1 USDA-Agricultural Research Service, San Joaquin Valley Agricultural Sciences Center, Parlier, CA, United States of America; 2 Cooperative Extension, Division of Agriculture and Natural Resources, University of California, Santa Rosa, CA, United States of America; University of Saskatchewan College of Agriculture and Bioresources, CANADA

## Abstract

*Spissistilus festinus* (Say) (Hemiptera: Membracidae) was shown to transmit Grapevine red blotch virus (GRBV) in a greenhouse study. Grapevines infected with GRBV exhibit reduced sugar accumulation, altered secondary metabolite production and delayed berry maturation that negatively impacts wine quality and economics. Augmentative biocontrol may be a useful integrated pest management (IPM) tool for suppressing *S*. *festinus* populations in vineyards, but minimal research has been conducted on testing potential predators against the different life stages of *S*. *festinus*. The susceptibility of *S*. *festinus* adults and nymphs (1^st^ through 5^th^ instar) to predation by six commercially available biocontrol agents in petri dish and bell bean plant arenas was determined under greenhouse conditions. No significant mortality of *S*. *festinus* nymphs or adults occurred when exposed to *Cryptolaemus montrouzieri* adults, *C*. *montrouzieri* larvae and *Sympherobius barberi* adults in petri dish or bell bean plant arenas. Significant mortality of 1^st^ and 2^nd^ instar nymphs of *S*. *festinus* in the presence of *Zelus renardii* nymphs was observed in petri dish but not in bell bean arenas. *Hippodamia convergens* adults and *Chrysoperla rufilabris* larvae both consumed a significant number of *S*. *festinus* nymphs in petri dish and bell bean arenas. No significant predation of *S*. *festinus* adults was documented in this experiment. Results of this study aid in identifying predators that may be suitable candidates for additional field testing to determine their potential efficacy as biocontrol agents of *S*. *festinus* in a vineyard setting.

## Introduction

Grapevine red blotch virus (GRBV) is the causative agent of grapevine red blotch disease (GRBD) [1–3]. Grapevines have tested positive for GRBV throughout the United States, Canada, and Mexico [4–6]. GRBD delays fruit maturity, reduces sugar accumulation in berries, and affects the production of secondary metabolites that contribute to color, aroma, and flavor of finished wine [7–10]. As a result, GRBD negatively impacts wine grape production due to the reduction in quality and commercial value of wine produced from infected grapes [11].

The threecornered alfalfa hopper, *Spissistilus festinus* (Say) (Hemiptera: Membracidae) was shown to transmit GRBV to grapevines in a greenhouse study [12]. Prior to this discovery, *S*. *festinus* was considered an incidental pest of grapevines [13] due to their characteristic girdling of petioles and shoots [14]. In fact, research on *S*. *festinus* has typically focused on other cropping systems such as alfalfa, peanuts, and soybeans [14,15], with little reported about the treehopper’s association with vineyards. Given the threat of GRBV transmission by *S*. *festinus* in grapevines, recent studies have focused on the biology and behavior of *S*. *festinus* in vineyards, on identifying feeding and reproductive hosts and documenting the treehopper’s seasonal dynamics in vineyards [16–18].

In northern California, overwintering *S*. *festinus* adults move into vineyards from surrounding habitats in early to mid-February. On arrival to a vineyard, females may oviposit on ground vegetation provided suitable hosts are available [18]. Legumes are preferred hosts of *S*. *festinus* [14,15,17,19] and are commonly planted as cover crops in vineyards or constitute part of the resident vegetation [20,21]. As springtime populations of *S*. *festinus* inhabit ground cover present in vineyards, insecticides are not a viable control option as candidate insecticides are not registered for application to vineyard ground cover. Instead, resident vegetation and/or cover crops could be tilled under during spring when early instars of *S*. *festinus* are present. While springtime tillage is a common vineyard practice [22,23], not all vineyard managers subscribe to disturbing the soil.

Some adult *S*. *festinus* will move into the grape canopy starting in June, causing girdling damage to grape petioles and shoots and potentially transmitting GRBV. While the number of *S*. *festinus* adults moving into the grape canopy increases as the season progresses, insecticide treatments applied to the grape canopy are expected to have limited efficacy as many *S*. *festinus* remain on ground vegetation [18]. In addition, Cieniewicz et al. [24] showed that *S*. *festinus* collected from vineyards do not test positive for GRBV until June, suggesting that management actions designed to suppress *S*. *festinus* populations should be implemented prior to June.

Release of biocontrol agents in vineyards is an additional IPM tactic that could be used to reduce *S*. *festinus* populations in vineyards. Previous tests by Medal et al. [25] evaluated predation of *S*. *festinus* nymphs and adults by *Geocoris punctipes*, *Nabis roseipennis*, and *Orius insidiosus*. However, these species only represent a few of the many predacious insects available for purchase from commercial insectaries. In this study, five additional predator species that are commercially available for purchase but have not been previously tested were evaluated. A greenhouse study was designed to determine the life stages of *S*. *festinus* most susceptible to predation by the following biological control agents: *Hippodamia convergens* (Guérin-Méneville) (Coleoptera: Coccinellidae) adults, *Cryptolaemus montrouzieri* (Mulsant) (Coleoptera: Coccinellidae) adults and 2^nd^ instar larvae, *Chrysoperla rufilabris* (Burmeister) (Neuroptera: Chrysopidae) 2^nd^ instar larvae, *Sympherobius barberi* (Banks) (Neuroptera: Hemerobiidae) adults, and *Zelus renardii* (Kolenati) (Hemiptera: Reduviidae) 1^st^ instar nymphs. Results from this study aid in identifying biological control agents that may be suitable for additional field testing, with the ultimate goal of assisting growers and vineyard managers in choosing appropriate biological control agents to suppress *S*. *festinus* populations which may aid in reducing vector-mediated spread of GRBV.

## Materials and methods

### Insect colony

An *S*. *festinus* colony was established in March 2019 from adults collected by sweep net from alfalfa, *Medicago sativa*, fields in Parlier, CA (36° 36' 11.9'' N, 119° 30' 37.7'' W, elevation 103 m) and Clovis, CA (36° 47' 39.9'' N, 119° 37' 55.0'' W, elevation 113 m). The authors have a permit issued from the California Department of Food and Agricultural for collecting *S*. *festinus* (permit #3479). *Spissistilus festinus* were collected from privately owned land with permission obtained from the owners prior to collecting. Field studies did not involve endangered or protected species. The colony was maintained in the greenhouse in insect rearing cages (Model BD2120, MegaView Science Co. Ltd., Taichung, Taiwan) and reared on a combination of potted bell bean (*Vicia faba*) and purple vetch (*Vicia benghalensis*). Field collected adults were occasionally added to the colony to increase genetic diversity. Adult and 1^st^-5^th^ instar nymphs of *S*. *festinus* were collected from the colony prior to setting up the assays. Eggs were not tested.

### Predators

Five species of biocontrol agents were tested: *Hippodamia convergens* (Guérin-Méneville) (Coleoptera: Coccinellidae), *Cryptolaemus montrouzieri* (Mulsant) (Coleoptera: Coccinellidae), *Chrysoperla rufilabris* (Burmeister) (Neuroptera: Chrysopidae), *Sympherobius barberi* (Banks) (Neuroptera: Hemerobiidae), and *Zelus renardii* (Kolenati) (Hemiptera: Reduviidae). *Hippodamia convergens* were collected by sweep net from alfalfa, *Medicago sativa*, fields in Clovis, CA (36° 47' 39.9'' N, 119° 37' 55.0'' W, elevation 113 m). All remaining predators were purchased from California based commercial biological control providers. The predator’s life stage chosen to be tested against the different life stages of *S*. *festinus* was dependent upon the life stage of the predator available for purchase from commercial insectaries. This decision was made to reflect the predator/prey interactions expected from the life stage supply available to industry purchasers. *Hippodamia convergens* and *S*. *barberi* were tested as adults, *C*. *montrouzieri* were tested separately as 2^nd^ instar larvae and adults, *C*. *rufilabris* were tested as 2^nd^ instar larvae, and *Z*. *renardii* were tested as 1^st^ instar nymphs. Upon delivery, all predators were held individually in 1-ounce condiment cups and starved for 24 hours prior to being introduced into their testing arena with the exception of *Z*. *renardii*, which were purchased as eggs and reared on *Aphis gossypii* for 4–7 days before being subjected to the same 24-hour starvation period prior to testing.

### Petri dish testing arena

To provide an *S*. *festinus* food source in petri dish arenas, green beans (*Phaseolus vulgaris*) were cut into ~2.5 cm sections and hot glued to the bottom of the Petri dish (100 mm x 15 mm) to hold the green bean section in place. A crumpled moistened paper towel (17.5 cm^2^) was placed in each Petri dish as a water source. One *S*. *festinus* of a certain life stage (1^st^ through 5^th^ instar or adult) was placed on the green bean in each petri dish and allowed 30 minutes to settle before a predator was introduced into the arena. Each Petri dish was sealed with Parafilm immediately following introduction of the predator. Petri dishes were placed in the greenhouse and *S*. *festinus* mortality recorded after 24 hours. Ten replicates of each of the six developmental stages (1^st^– 5^th^ instar and adult) of *S*. *festinus* were tested. To quantify *S*. *festinus* background mortality, five Petri dishes per *S*. *festinus* life stage that contained food and a water source, but no predator served as controls.

### Bell bean testing cage

As petri dish arenas are a simplified environment, additional tests were conducted with *S*. *festinus* held on plants. Bell beans (*Vicia faba*) were chosen as the test plant because they are a feeding host of *S*. *festinus* (Preto et al. 2018b) and have an upright growth pattern. Bell bean seeds were purchased from Harmony Farms Supply & Nursery, Sebastopol, CA, USA. Professional growing mix potting soil was purchased from Sun Gro Horticulture Canada Ltd., Seba Beach, Canada. Dart red plastic party cups 532-ml (18GR20, Dart Container Corporation, Mason, MI) served as pots with holes (~4 mm) drilled in the bottom for drainage. Each pot was planted with one bell bean seed and allowed to grow for 12–14 days to ~10–15 cm in height before being used for testing.

Mesh cages were constructed by cutting 3.8 liter plain top paint strainers (#31101, Trimaco, Morrisville, NC, USA) in half vertically and sealing the length of the vertical opening with hot glue. Bamboo skewers– 46 cm (#3166, Chef’s Fun Foods, East Brunswick, NJ, USA) were cut to 36.8 cm and three skewers were placed vertically into the pots equidistant from each other in a triangular arrangement. Mesh cages were placed over the skewers. One *S*. *festinus* of a certain life stage was placed on the base of the bell bean plant and allowed 30 minutes to settle before a predator was introduced into the cage. Each cage was sealed and attached to the pot with 5.1 cm packaging tape (S-423, Uline, Pleasant Prairie, WI, USA) immediately following introduction of the predator. Bell bean cages were held in the greenhouse and mortality recorded after 24 hours. Ten replicates each of six developmental life stages (1^st^– 5^th^ instar and adult) of *S*. *festinus* were tested. Ten bell bean cages per life stage containing prey but no predator served as controls that quantified background mortality. Petri dish and bell bean arenas were conducted simultaneously for each predator tested.

### Data analysis

A generalized linear model with binomial error and a logit link function was fit to control and predator data sets using JMP stats [26]. Each analysis tested effects of predator species, *S*. *festinus* life stage, test arena type, and all possible interaction terms on *S*. *festinus* mortality. As predators were not present in controls, the predator species term in the analysis of data collected from controls represented the effect of test date on *S*. *festinus* mortality. Replicates in which the predator died before the end of the assay were excluded from analysis (predators died in 43 out of 360 trials in petri dishes [11.9%] and 18 out of 360 trials on bell bean plants [5%]).

To determine the predator species by *S*. *festinus* life stage combinations that resulted in significant *S*. *festinus* mortality, adjusted mortality was determined for each predator species by *S*. *festinus* life stage combination using Abott’s formula [27]. Subsequently, 95 percent confidence intervals were determined for the adjusted mortality rate. If adjusted mortality for a specific predator species and life stage combination was positive and the lower bound of the 95% confidence interval did not overlap with zero, *S*. *festinus* mortality in the presence of the predator was considered significant. Adjusted mortality values of 100% or 0% were not associated with an estimate of error as there was no variation in the response (i.e., either all prey were eaten or all prey survived).

## Results

### *S*. *festinus* mortality depended on predator species and life stage exposed to predation

Eight out of 172 (4.7%) *S*. *festinus* held in control petri dishes (i.e., in the absence of predators) died and 30 out of 360 (8.3%) *S*. *festinus* held on control bell bean plants died. In controls, *S*. *festinus* mortality was not affected by predator species, *S*. *festinus* life stage, test arena (petri dish versus bell bean) or any of the interaction terms (Table 1). As *S*. *festinus* held in control treatments were not in the presence of predators, the predator species term in the analysis represented the effect of test date. Analysis of *S*. *festinus* mortality data in the presence of predators, indicated a significant predator species by *S*. *festinus* life stage interaction (Table 1). Thus, *S*. *festinus* mortality depended on the specific combination of predator species and *S*. *festinus* life stage.

**Table 1:** Results of generalized linear models with binomial error that tested for effects of predator species, prey life stage, test type, and all interaction terms on *Spissistilus festinus* mortality in control treatments (no predators) and in predator treatments. (P ≤ 0.05).

		Controls (no predators)		Treatments (with predators)	
Source	DF	Χ^2^	P	Χ^2^	P
Predator species^a^	5	0.6	0.99	0.0	1.0
Prey life stage	5	0.0	1.0	0.0	1.0
Test type (petri dish versus bell bean)	1	0.0	1.0	3.4	0.07
Predator species x prey life stage	25	0.0	1.0	99.9	<0.0001
Predator species x test type	5	0.0	1.0	0.0	1.0
Prey life stage x test type	5	0.0	1.0	0.0	1.0
Predator species x prey life stage x test type	25	0.0	1.0	3.2	1.0

^a^ As predators were not present in control treatments, the “predator species” term in the analysis of the control data set represents the effect of setup date.

### *C*. *montrouzieri* and *S*. *barberi* did not consume *S*. *festinus*

**Fig 1:**
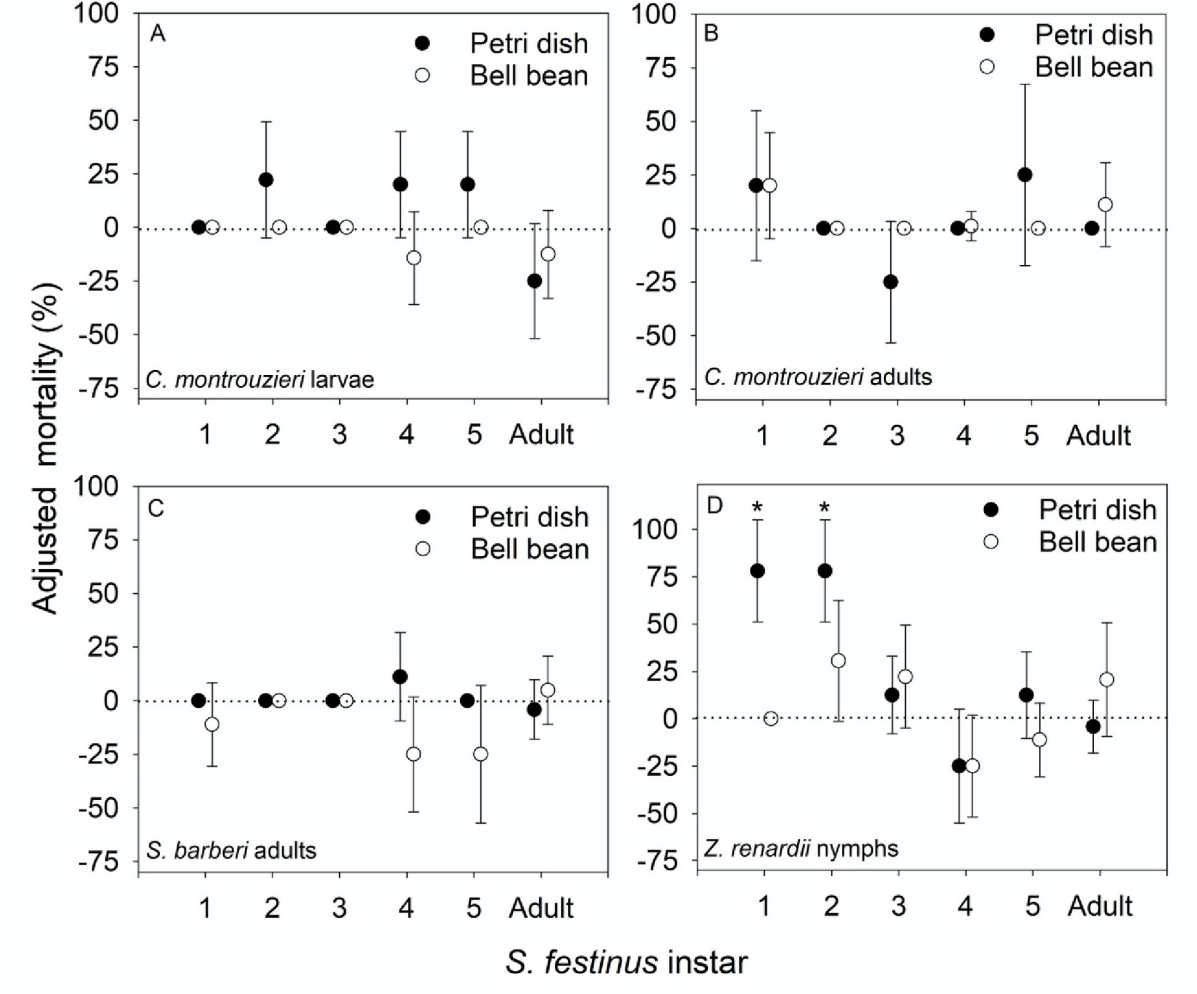
Results of petri dish and bell bean bioassays using A) *C*. *montrouzieri* larvae, B) *C*. *montrouzieri* adults, C) *S*. *barberi* adults, and D) *Z*. *renardii* nymphs. The percentage of prey that were killed in predator treatments was adjusted using Abott’s formula to account for background mortality observed in controls. Error bars represent 95% confidence intervals. Mortality was considered significant if it was positive and the confidence interval did not overlap with zero (indicated by an asterisk). Negative adjusted mortality values indicate greater mortality in controls than in predator treatments.

In petri dish and bell bean arenas, *C*. *montrouzieri* larvae (Fig 1A) and adults (Fig 1B) did not consume *S*. *festinus* at a rate sufficient to distinguish from background mortality in control arenas. Likewise, *S*. *barberi* adults did not consume *S*. *festinus* in petri dish or bell bean arenas (Fig 1C).

### *Z*. *renardii* consumed early instar *S*. *festinus* in petri dish arenas, but not in bell bean arenas

In petri dish arenas, presence of *Z*. *renardii* nymphs resulted in significant morality of 1^st^ and 2^nd^ instar *S*. *festinus*, although *Z*. *renardii* nymphs did not signicantly consume any of the other *S*. *festinus* life stages tested (Fig 1D). In bell bean arenas, *S*. *festinus* mortality in the presence of *Z*. *renardii* was not distinguishable from background mortality observed in controls for all life stages tested (Fig 1D). While *Z*. *renardii* nymphs appeared to consume some life stages of *S*. *festinus*, absence of significant *S*. *festinus* mortality on bell beans suggests that *Z*. *renardii* may have had greater difficulty locating *S*. *festinus* nymphs in bell bean arenas than in petri dish arenas.

### *H*. *convergens* and *C*. *rufilabris* consumed *S*. *festinus* in petri dish and bell bean arenas

**Fig 2:**
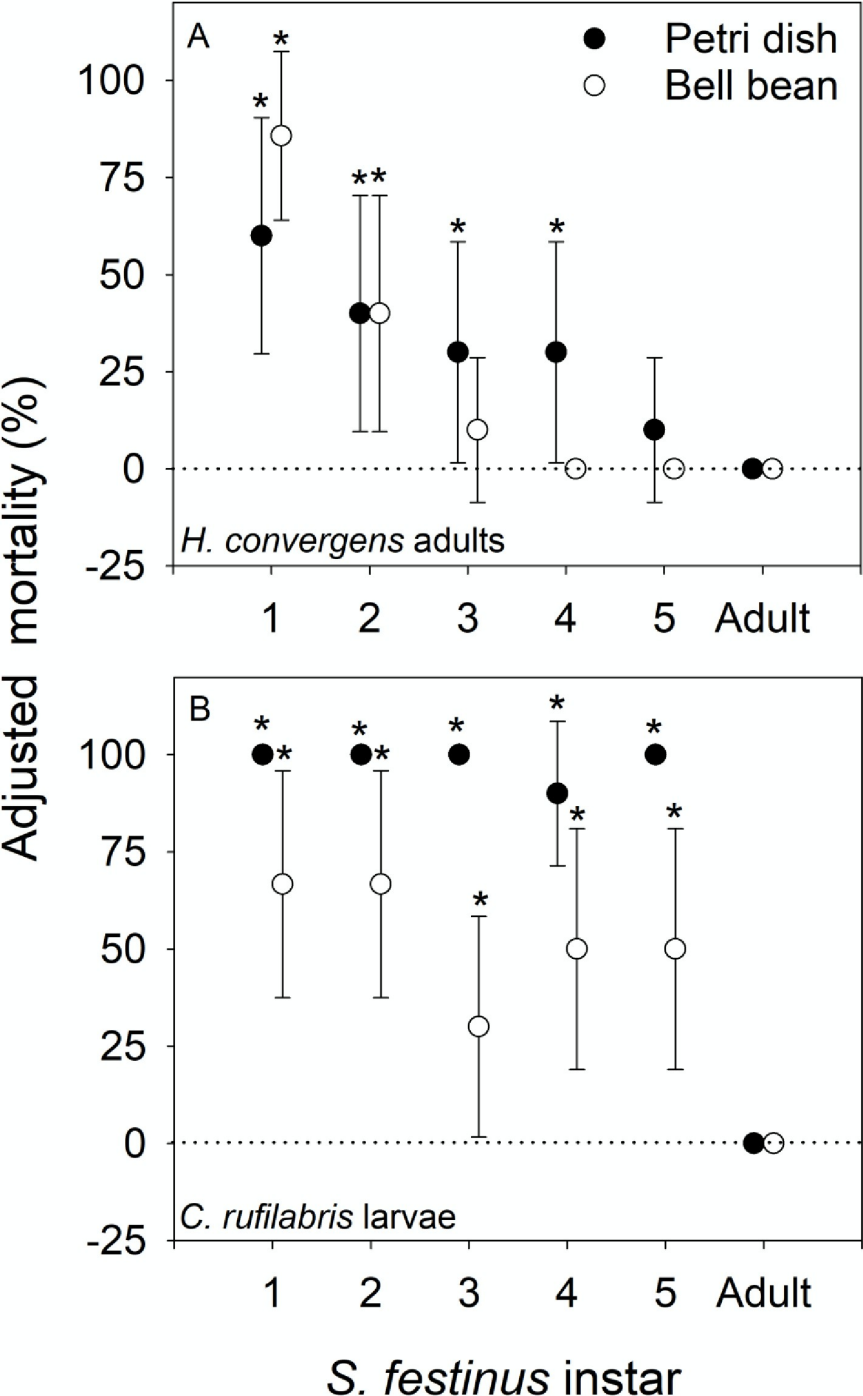
Results of petri dish and bell bean bioassays using A) *H*. *convergens* adults and B) *C*. *rufilabris* larvae. The percentage of prey that were killed in predator treatments was adjusted using Abott’s formula to account for background mortality observed in controls. Error bars represent 95% confidence intervals. Mortality was considered significant if it was positive and the confidence interval did not overlap with zero (indicated by an asterisk). Negative adjusted mortality values indicate greater mortality in controls than in predator treatments.

In petri dish arenas, *H*. *convergens* adults caused significant mortality of 1^st^ through 4^th^ instar *S*. *festinus* nymphs (Fig 2A). However, in bell bean arenas, *H*. *convergens* adults caused significant mortality to only 1^st^ and 2^nd^ instar *S*. *festinus* (Fig 2A). Finally, *C*. *rufilabris* caused significant mortality of 1^st^ through 5^th^ instar *S*. *festinus* nymphs in petri dish and bell bean arenas (Fig 2B). Mortality of *S*. *festinus* adults by all six predators tested in this experiment was not significant (Figs 1 and 2).

## Discussion

The petri dish and bell bean arenas were no-choice assays in which each biocontrol agent was confined to their assigned environment and could either attack/consume the prey provided or not. Predators were unable to choose a different environment or alternate feeding option. The petri dish arena was the most artificial constituting a small area for the predator to search without refuge for the prey. In contrast, the bell bean arena more closely resembled a natural environment with a host plant, soil, and areas for refuge. As petri dish arenas were simplified environments compared to bell bean arenas, petri dish arenas may overestimate the potential of the predator to serve as a biocontrol agent. While bell bean arenas provided a more complex environment, bell bean arenas were still limited in size, confined the prey in proximity to the predator, and lacked alternative prey/predators that may be found in an actual vineyard setting. Nonetheless, both tests aid in determining which (if any) *S*. *festinus* life stages may be within the predator’s host range and may be suitable for additional testing. Results indicate that *C*. *montrouzieri* larvae and adults and *S*. *barberi* adults were not effective predators of the six developmental life stages (1-5^th^ instar and adults) of *S*. *festinus*. In contrast, *Z*. *renardii* nymphs, *H*. *convergens* adults, and *C*. *rufilabris* larvae attacked some life stages of *S*. *festinus*. None of the tested predators consumed *S*. *festinus* adults.

In petri dish arenas, *Z*. *renardii* nymphs significantly increased mortality of 1^st^ and 2^nd^ instar *S*. *festinus* (Fig 1D) but did not cause significant mortality of larger instar or adult *S*. *festinus*. It is possible that the relatively large size of late instar *S*. *festinus* exceeded the handling capacity of the relatively small 1^st^ instar *Z*. *renardii*. This hypothesis is supported by the results of Fye [28] which showed that later instars of *Z*. *renardii* were more aggressive and had better searching capability and prey interception than early instars. As a result, it is possible that trials using larger *Z*. *renardii* instars might yield different results. While significant predation of 1^st^ and 2^nd^ instar *S*. *festinus* by *Z*. *renardii* nymphs was observed in petri dish arenas, no significant mortality due to predation was observed in bell bean arenas (Fig 1D). Significant mortality of early *S*. *festinus* instars in petri dish arenas but not in bell bean plant arenas indicates that *S*. *festinus* is within the host range of *Z*. *renardii* but the searching capacity required to find early instar prey in bell bean cages was not sufficient in 1^st^ instar *Z*. *renardii* nymphs.

Significant mortality of *S*. *festinus* by *H*. *convergens* adults was documented for 1^st^ through 4^th^ instars in petri dish arenas (Fig 2A). In bell bean arenas (Fig 2A), *H*. *convergens* predation was limited to 1^st^ and 2^nd^ instars of *S*. *festinus* that were similar in size to aphids, the preferred prey of *H*. *convergens* [29]. If *H*. *convergens* adults were used in an augmentive biological control program, field releases would need to coincide with presence of early instar *S*. *festinus* in field populations. Timing such releases may be challenging as early instar *S*. *festinus* nymphs feed at the base of plants, where they are often not observed using common sampling methods such as sweep netting [18]. In addition, the behavior of adult *H*. *convergens* must also be taken into consideration prior to field testing and/or release of *H*. *convergens* adults as a biological control agent in vineyards. Many commercial insectaries acquire adult *H*. *convergens* by collecting them from aggregations found in the foothills of the Sierra Nevada mountain range in California. In many cases, the innate dispersal behavior of *H*. *convergens* has prevented it from being successfully used in large-scale biocontrol releases [29,30]. Finally, *H*. *convergens* adults exhibit aggregation behaviors [29,30] and if large populations were to subsist in the vineyard during grape harvest, when crushed, *H*. *convergens* can impart a combination of methoxypyrazines that result in a wine flaw referred to as Ladybird taint [31,32].

Larvae of *C*. *rufilabris* caused significant mortality of 1^st^ through 5^th^ instar *S*. *festinus* in petri dish and bell bean arenas (Fig 2B). These results suggest that *C*. *rufilabris* larvae are ideal candidates for additional testing as part of an *S*. *festinus* biological control program for several reasons. First, testing here demonstrates that *S*. *festinus* is within the host range of *C*. *rufilabris* and that *C*. *rufilabris* can attack all nymphal stages of *S*. *festinus*. Second, as *C*. *rufilabris* may be purchased as larvae, there is no risk of dispersal from the release habitat. Finally, *Chrysoperla* sp. are known to consume other vineyard pests such as the leafhoppers *Erythroneura variabilis* (Beamer) and *E*. *elegantula* (Osborn) [33]. Thus, release of *C*. *rufilabris* may aid in suppressing vineyard pests other than *S*. *festinus*.

The use of augmentative biocontrol to help reduce an insect pest population is an important tool that can be utilized as part of an IPM program. Identifying commercially available predators that may assist in reducing *S*. *festinus* populations is promising, especially in no-till vineyards where *S*. *festinus* is present. Results from this study suggest that *C*. *rufilabris* may be an ideal candidate for augmentative releases. Future research involving choice tests and measuring predation rates as well as field testing of *C*. *rufilabris* releases in a vineyard setting and the subsequent impact on *S*. *festinus* populations along with the spread of GRBD are needed.

## Supporting information

S1 Table Raw data evaluating predation of the threecorned alfalfa hopper by six predators. (DOCX)
