# Supplementary material for: Spissistilus festinus (Hemiptera: Membracidae) susceptibility to six generalist predators
Source: PLoS One. 2020 Nov 30;15(11):e0242775. doi: 10.1371/journal.pone.0242775 (PMC7703964; doi:10.1371/journal.pone.0242775)
Supplement: S1 Table — (DOCX) [file pone.0242775.s001.docx]

Table S1. Raw data evaluating predation of the threecorned alfalfa hopper by six predators.

| Predator species | Prey life stage | test type | rep | test or control | prey dead = 1 | predator dead = 1 |
| --- | --- | --- | --- | --- | --- | --- |
| H. convergens - a | 1 | petri dish | 1 | test | 1 | 0 |
| H. convergens - a | 1 | petri dish | 2 | test | 0 | 0 |
| H. convergens - a | 1 | petri dish | 3 | test | 0 | 0 |
| H. convergens - a | 1 | petri dish | 4 | test | 1 | 0 |
| H. convergens - a | 1 | petri dish | 5 | test | 1 | 0 |
| H. convergens - a | 1 | petri dish | 6 | test | 0 | 0 |
| H. convergens - a | 1 | petri dish | 7 | test | 1 | 0 |
| H. convergens - a | 1 | petri dish | 8 | test | 0 | 0 |
| H. convergens - a | 1 | petri dish | 9 | test | 1 | 0 |
| H. convergens - a | 1 | petri dish | 10 | test | 1 | 0 |
| H. convergens - a | 1 | petri dish | 1 | control | 0 | 0 |
| H. convergens - a | 1 | petri dish | 2 | control | 0 | 0 |
| H. convergens - a | 1 | petri dish | 3 | control | 0 | 0 |
| H. convergens - a | 1 | petri dish | 4 | control | 0 | 0 |
| H. convergens - a | 1 | petri dish | 5 | control | 0 | 0 |
| H. convergens - a | 2 | petri dish | 1 | test | 0 | 0 |
| H. convergens - a | 2 | petri dish | 2 | test | 0 | 0 |
| H. convergens - a | 2 | petri dish | 3 | test | 1 | 0 |
| H. convergens - a | 2 | petri dish | 4 | test | 0 | 0 |
| H. convergens - a | 2 | petri dish | 5 | test | 0 | 0 |
| H. convergens - a | 2 | petri dish | 6 | test | 0 | 0 |
| H. convergens - a | 2 | petri dish | 7 | test | 1 | 0 |
| H. convergens - a | 2 | petri dish | 8 | test | 1 | 0 |
| H. convergens - a | 2 | petri dish | 9 | test | 0 | 0 |
| H. convergens - a | 2 | petri dish | 10 | test | 1 | 0 |
| H. convergens - a | 2 | petri dish | 1 | control | 0 | 0 |
| H. convergens - a | 2 | petri dish | 2 | control | 0 | 0 |
| H. convergens - a | 2 | petri dish | 3 | control | 0 | 0 |
| H. convergens - a | 2 | petri dish | 4 | control | 0 | 0 |
| H. convergens - a | 2 | petri dish | 5 | control | 0 | 0 |
| H. convergens - a | 3 | petri dish | 1 | test | 0 | 0 |
| H. convergens - a | 3 | petri dish | 2 | test | 0 | 0 |
| H. convergens - a | 3 | petri dish | 3 | test | 1 | 0 |
| H. convergens - a | 3 | petri dish | 4 | test | 0 | 0 |
| H. convergens - a | 3 | petri dish | 5 | test | 0 | 0 |
| H. convergens - a | 3 | petri dish | 6 | test | 0 | 0 |
| H. convergens - a | 3 | petri dish | 7 | test | 0 | 0 |
| H. convergens - a | 3 | petri dish | 8 | test | 0 | 0 |
| H. convergens - a | 3 | petri dish | 9 | test | 1 | 0 |
| H. convergens - a | 3 | petri dish | 10 | test | 1 | 0 |
| H. convergens - a | 3 | petri dish | 1 | control | 0 | 0 |
| H. convergens - a | 3 | petri dish | 2 | control | 0 | 0 |
| H. convergens - a | 3 | petri dish | 3 | control | 0 | 0 |
| H. convergens - a | 3 | petri dish | 4 | control | 0 | 0 |
| H. convergens - a | 3 | petri dish | 5 | control | 0 | 0 |
| H. convergens - a | 4 | petri dish | 1 | test | 0 | 0 |
| H. convergens - a | 4 | petri dish | 2 | test | 0 | 0 |
| H. convergens - a | 4 | petri dish | 3 | test | 1 | 0 |
| H. convergens - a | 4 | petri dish | 4 | test | 0 | 0 |
| H. convergens - a | 4 | petri dish | 5 | test | 0 | 0 |
| H. convergens - a | 4 | petri dish | 6 | test | 0 | 0 |
| H. convergens - a | 4 | petri dish | 7 | test | 1 | 0 |
| H. convergens - a | 4 | petri dish | 8 | test | 1 | 0 |
| H. convergens - a | 4 | petri dish | 9 | test | 0 | 0 |
| H. convergens - a | 4 | petri dish | 10 | test | 0 | 0 |
| H. convergens - a | 4 | petri dish | 1 | control | 0 | 0 |
| H. convergens - a | 4 | petri dish | 2 | control | 0 | 0 |
| H. convergens - a | 4 | petri dish | 3 | control | 0 | 0 |
| H. convergens - a | 4 | petri dish | 4 | control | 0 | 0 |
| H. convergens - a | 4 | petri dish | 5 | control | 0 | 0 |
| H. convergens - a | 5 | petri dish | 1 | test | 0 | 0 |
| H. convergens - a | 5 | petri dish | 2 | test | 0 | 0 |
| H. convergens - a | 5 | petri dish | 3 | test | 0 | 0 |
| H. convergens - a | 5 | petri dish | 4 | test | 0 | 0 |
| H. convergens - a | 5 | petri dish | 5 | test | 0 | 0 |
| H. convergens - a | 5 | petri dish | 6 | test | 0 | 0 |
| H. convergens - a | 5 | petri dish | 7 | test | 0 | 0 |
| H. convergens - a | 5 | petri dish | 8 | test | 0 | 0 |
| H. convergens - a | 5 | petri dish | 9 | test | 1 | 0 |
| H. convergens - a | 5 | petri dish | 10 | test | 0 | 0 |
| H. convergens - a | 5 | petri dish | 1 | control | 0 | 0 |
| H. convergens - a | 5 | petri dish | 2 | control | 0 | 0 |
| H. convergens - a | 5 | petri dish | 3 | control | 0 | 0 |
| H. convergens - a | 5 | petri dish | 4 | control | 0 | 0 |
| H. convergens - a | 5 | petri dish | 5 | control | 0 | 0 |
| H. convergens - a | a | petri dish | 1 | test | 0 | 0 |
| H. convergens - a | a | petri dish | 2 | test | 0 | 0 |
| H. convergens - a | a | petri dish | 3 | test | 0 | 0 |
| H. convergens - a | a | petri dish | 4 | test | 0 | 0 |
| H. convergens - a | a | petri dish | 5 | test | 0 | 0 |
| H. convergens - a | a | petri dish | 6 | test | 0 | 0 |
| H. convergens - a | a | petri dish | 7 | test | 0 | 0 |
| H. convergens - a | a | petri dish | 8 | test | 0 | 0 |
| H. convergens - a | a | petri dish | 9 | test | 0 | 0 |
| H. convergens - a | a | petri dish | 10 | test | 0 | 0 |
| H. convergens - a | a | petri dish | 1 | control | 0 | 0 |
| H. convergens - a | a | petri dish | 2 | control | 0 | 0 |
| H. convergens - a | a | petri dish | 3 | control | 0 | 0 |
| H. convergens - a | a | petri dish | 4 | control | 0 | 0 |
| H. convergens - a | a | petri dish | 5 | control | 0 | 0 |
| H. convergens - a | 1 | bell bean | 1 | test | 1 | 0 |
| H. convergens - a | 1 | bell bean | 2 | test | 1 | 0 |
| H. convergens - a | 1 | bell bean | 3 | test | 1 | 0 |
| H. convergens - a | 1 | bell bean | 4 | test | 1 | 0 |
| H. convergens - a | 1 | bell bean | 5 | test | 1 | 0 |
| H. convergens - a | 1 | bell bean | 6 | test | 1 | 0 |
| H. convergens - a | 1 | bell bean | 7 | test | 1 | 0 |
| H. convergens - a | 1 | bell bean | 8 | test | 1 | 0 |
| H. convergens - a | 1 | bell bean | 9 | test | 0 | 0 |
| H. convergens - a | 1 | bell bean | 10 | test | 1 | 0 |
| H. convergens - a | 1 | bell bean | 1 | control | 1 | 0 |
| H. convergens - a | 1 | bell bean | 2 | control | 0 | 0 |
| H. convergens - a | 1 | bell bean | 3 | control | 0 | 0 |
| H. convergens - a | 1 | bell bean | 4 | control | 0 | 0 |
| H. convergens - a | 1 | bell bean | 5 | control | 1 | 0 |
| H. convergens - a | 1 | bell bean | 6 | control | 1 | 0 |
| H. convergens - a | 1 | bell bean | 7 | control | 0 | 0 |
| H. convergens - a | 1 | bell bean | 8 | control | 0 | 0 |
| H. convergens - a | 1 | bell bean | 9 | control | 0 | 0 |
| H. convergens - a | 1 | bell bean | 10 | control | 0 | 0 |
| H. convergens - a | 2 | bell bean | 1 | test | 1 | 0 |
| H. convergens - a | 2 | bell bean | 2 | test | 1 | 0 |
| H. convergens - a | 2 | bell bean | 3 | test | 1 | 0 |
| H. convergens - a | 2 | bell bean | 4 | test | 0 | 0 |
| H. convergens - a | 2 | bell bean | 5 | test | 0 | 0 |
| H. convergens - a | 2 | bell bean | 6 | test | 0 | 0 |
| H. convergens - a | 2 | bell bean | 7 | test | 0 | 0 |
| H. convergens - a | 2 | bell bean | 8 | test | 0 | 0 |
| H. convergens - a | 2 | bell bean | 9 | test | 0 | 0 |
| H. convergens - a | 2 | bell bean | 10 | test | 1 | 0 |
| H. convergens - a | 2 | bell bean | 1 | control | 0 | 0 |
| H. convergens - a | 2 | bell bean | 2 | control | 0 | 0 |
| H. convergens - a | 2 | bell bean | 3 | control | 0 | 0 |
| H. convergens - a | 2 | bell bean | 4 | control | 0 | 0 |
| H. convergens - a | 2 | bell bean | 5 | control | 0 | 0 |
| H. convergens - a | 2 | bell bean | 6 | control | 0 | 0 |
| H. convergens - a | 2 | bell bean | 7 | control | 0 | 0 |
| H. convergens - a | 2 | bell bean | 8 | control | 0 | 0 |
| H. convergens - a | 2 | bell bean | 9 | control | 0 | 0 |
| H. convergens - a | 2 | bell bean | 10 | control | 0 | 0 |
| H. convergens - a | 3 | bell bean | 1 | test | 0 | 0 |
| H. convergens - a | 3 | bell bean | 2 | test | 0 | 0 |
| H. convergens - a | 3 | bell bean | 3 | test | 0 | 0 |
| H. convergens - a | 3 | bell bean | 4 | test | 0 | 0 |
| H. convergens - a | 3 | bell bean | 5 | test | 0 | 0 |
| H. convergens - a | 3 | bell bean | 6 | test | 0 | 0 |
| H. convergens - a | 3 | bell bean | 7 | test | 0 | 0 |
| H. convergens - a | 3 | bell bean | 8 | test | 0 | 0 |
| H. convergens - a | 3 | bell bean | 9 | test | 0 | 0 |
| H. convergens - a | 3 | bell bean | 10 | test | 1 | 0 |
| H. convergens - a | 3 | bell bean | 1 | control | 0 | 0 |
| H. convergens - a | 3 | bell bean | 2 | control | 0 | 0 |
| H. convergens - a | 3 | bell bean | 3 | control | 0 | 0 |
| H. convergens - a | 3 | bell bean | 4 | control | 0 | 0 |
| H. convergens - a | 3 | bell bean | 5 | control | 0 | 0 |
| H. convergens - a | 3 | bell bean | 6 | control | 0 | 0 |
| H. convergens - a | 3 | bell bean | 7 | control | 0 | 0 |
| H. convergens - a | 3 | bell bean | 8 | control | 0 | 0 |
| H. convergens - a | 3 | bell bean | 9 | control | 0 | 0 |
| H. convergens - a | 3 | bell bean | 10 | control | 0 | 0 |
| H. convergens - a | 4 | bell bean | 1 | test | 0 | 0 |
| H. convergens - a | 4 | bell bean | 2 | test | 0 | 0 |
| H. convergens - a | 4 | bell bean | 3 | test | 0 | 0 |
| H. convergens - a | 4 | bell bean | 4 | test | 0 | 0 |
| H. convergens - a | 4 | bell bean | 5 | test | 0 | 0 |
| H. convergens - a | 4 | bell bean | 6 | test | 0 | 0 |
| H. convergens - a | 4 | bell bean | 7 | test | 0 | 0 |
| H. convergens - a | 4 | bell bean | 8 | test | 0 | 0 |
| H. convergens - a | 4 | bell bean | 9 | test | 0 | 0 |
| H. convergens - a | 4 | bell bean | 10 | test | 0 | 0 |
| H. convergens - a | 4 | bell bean | 1 | control | 0 | 0 |
| H. convergens - a | 4 | bell bean | 2 | control | 0 | 0 |
| H. convergens - a | 4 | bell bean | 3 | control | 0 | 0 |
| H. convergens - a | 4 | bell bean | 4 | control | 0 | 0 |
| H. convergens - a | 4 | bell bean | 5 | control | 0 | 0 |
| H. convergens - a | 4 | bell bean | 6 | control | 0 | 0 |
| H. convergens - a | 4 | bell bean | 7 | control | 0 | 0 |
| H. convergens - a | 4 | bell bean | 8 | control | 0 | 0 |
| H. convergens - a | 4 | bell bean | 9 | control | 0 | 0 |
| H. convergens - a | 4 | bell bean | 10 | control | 0 | 0 |
| H. convergens - a | 5 | bell bean | 1 | test | 0 | 0 |
| H. convergens - a | 5 | bell bean | 2 | test | 0 | 0 |
| H. convergens - a | 5 | bell bean | 3 | test | 0 | 0 |
| H. convergens - a | 5 | bell bean | 4 | test | 0 | 0 |
| H. convergens - a | 5 | bell bean | 5 | test | 0 | 0 |
| H. convergens - a | 5 | bell bean | 6 | test | 0 | 0 |
| H. convergens - a | 5 | bell bean | 7 | test | 0 | 0 |
| H. convergens - a | 5 | bell bean | 8 | test | 0 | 0 |
| H. convergens - a | 5 | bell bean | 9 | test | 0 | 0 |
| H. convergens - a | 5 | bell bean | 10 | test | 0 | 0 |
| H. convergens - a | 5 | bell bean | 1 | control | 0 | 0 |
| H. convergens - a | 5 | bell bean | 2 | control | 0 | 0 |
| H. convergens - a | 5 | bell bean | 3 | control | 0 | 0 |
| H. convergens - a | 5 | bell bean | 4 | control | 0 | 0 |
| H. convergens - a | 5 | bell bean | 5 | control | 0 | 0 |
| H. convergens - a | 5 | bell bean | 6 | control | 0 | 0 |
| H. convergens - a | 5 | bell bean | 7 | control | 0 | 0 |
| H. convergens - a | 5 | bell bean | 8 | control | 0 | 0 |
| H. convergens - a | 5 | bell bean | 9 | control | 0 | 0 |
| H. convergens - a | 5 | bell bean | 10 | control | 0 | 0 |
| H. convergens - a | a | bell bean | 1 | test | 0 | 0 |
| H. convergens - a | a | bell bean | 2 | test | 0 | 0 |
| H. convergens - a | a | bell bean | 3 | test | 0 | 0 |
| H. convergens - a | a | bell bean | 4 | test | 0 | 0 |
| H. convergens - a | a | bell bean | 5 | test | 0 | 0 |
| H. convergens - a | a | bell bean | 6 | test | 0 | 0 |
| H. convergens - a | a | bell bean | 7 | test | 0 | 0 |
| H. convergens - a | a | bell bean | 8 | test | 0 | 0 |
| H. convergens - a | a | bell bean | 9 | test | 0 | 0 |
| H. convergens - a | a | bell bean | 10 | test | 0 | 0 |
| H. convergens - a | a | bell bean | 1 | control | 0 | 0 |
| H. convergens - a | a | bell bean | 2 | control | 0 | 0 |
| H. convergens - a | a | bell bean | 3 | control | 0 | 0 |
| H. convergens - a | a | bell bean | 4 | control | 0 | 0 |
| H. convergens - a | a | bell bean | 5 | control | 0 | 0 |
| H. convergens - a | a | bell bean | 6 | control | 0 | 0 |
| H. convergens - a | a | bell bean | 7 | control | 0 | 0 |
| H. convergens - a | a | bell bean | 8 | control | 0 | 0 |
| H. convergens - a | a | bell bean | 9 | control | 0 | 0 |
| H. convergens - a | a | bell bean | 10 | control | 0 | 0 |
| C. montrouzieri - a | 1 | petri dish | 1 | test | 0 | 0 |
| C. montrouzieri - a | 1 | petri dish | 2 | test | 0 | 1 |
| C. montrouzieri - a | 1 | petri dish | 3 | test | 0 | 0 |
| C. montrouzieri - a | 1 | petri dish | 4 | test | 0 | 0 |
| C. montrouzieri - a | 1 | petri dish | 5 | test | 0 | 1 |
| C. montrouzieri - a | 1 | petri dish | 6 | test | 0 | 1 |
| C. montrouzieri - a | 1 | petri dish | 7 | test | 1 | 0 |
| C. montrouzieri - a | 1 | petri dish | 8 | test | 0 | 1 |
| C. montrouzieri - a | 1 | petri dish | 9 | test | 0 | 0 |
| C. montrouzieri - a | 1 | petri dish | 10 | test | 0 | 1 |
| C. montrouzieri - a | 1 | petri dish | 1 | control | 0 | 0 |
| C. montrouzieri - a | 1 | petri dish | 2 | control | 0 | 0 |
| C. montrouzieri - a | 1 | petri dish | 3 | control | 0 | 0 |
| C. montrouzieri - a | 1 | petri dish | 4 | control | 0 | 0 |
| C. montrouzieri - a | 1 | petri dish | 5 | control | 0 | 0 |
| C. montrouzieri - a | 2 | petri dish | 1 | test | 0 | 1 |
| C. montrouzieri - a | 2 | petri dish | 2 | test | 0 | 1 |
| C. montrouzieri - a | 2 | petri dish | 3 | test | 0 | 0 |
| C. montrouzieri - a | 2 | petri dish | 4 | test | 0 | 1 |
| C. montrouzieri - a | 2 | petri dish | 5 | test | 0 | 0 |
| C. montrouzieri - a | 2 | petri dish | 6 | test | 0 | 1 |
| C. montrouzieri - a | 2 | petri dish | 7 | test | 0 | 0 |
| C. montrouzieri - a | 2 | petri dish | 8 | test | 0 | 0 |
| C. montrouzieri - a | 2 | petri dish | 9 | test | 0 | 0 |
| C. montrouzieri - a | 2 | petri dish | 10 | test | 0 | 0 |
| C. montrouzieri - a | 2 | petri dish | 1 | control | 0 | 0 |
| C. montrouzieri - a | 2 | petri dish | 2 | control | 0 | 0 |
| C. montrouzieri - a | 2 | petri dish | 3 | control | 0 | 0 |
| C. montrouzieri - a | 2 | petri dish | 4 | control | 0 | 0 |
| C. montrouzieri - a | 2 | petri dish | 5 | control | 0 | 0 |
| C. montrouzieri - a | 3 | petri dish | 1 | test | 0 | 0 |
| C. montrouzieri - a | 3 | petri dish | 2 | test | 0 | 0 |
| C. montrouzieri - a | 3 | petri dish | 3 | test | 0 | 0 |
| C. montrouzieri - a | 3 | petri dish | 4 | test | 0 | 1 |
| C. montrouzieri - a | 3 | petri dish | 5 | test | 0 | 0 |
| C. montrouzieri - a | 3 | petri dish | 6 | test | 0 | 0 |
| C. montrouzieri - a | 3 | petri dish | 7 | test | 0 | 0 |
| C. montrouzieri - a | 3 | petri dish | 8 | test | 0 | 0 |
| C. montrouzieri - a | 3 | petri dish | 9 | test | 0 | 0 |
| C. montrouzieri - a | 3 | petri dish | 10 | test | 0 | 0 |
| C. montrouzieri - a | 3 | petri dish | 1 | control | 0 | 0 |
| C. montrouzieri - a | 3 | petri dish | 2 | control | 0 | 0 |
| C. montrouzieri - a | 3 | petri dish | 3 | control | 0 | 0 |
| C. montrouzieri - a | 3 | petri dish | 4 | control | 0 | 0 |
| C. montrouzieri - a | 3 | petri dish | 5 | control | 1 | 0 |
| C. montrouzieri - a | 4 | petri dish | 1 | test | 0 | 1 |
| C. montrouzieri - a | 4 | petri dish | 2 | test | 0 | 0 |
| C. montrouzieri - a | 4 | petri dish | 3 | test | 0 | 0 |
| C. montrouzieri - a | 4 | petri dish | 4 | test | 0 | 0 |
| C. montrouzieri - a | 4 | petri dish | 5 | test | 0 | 1 |
| C. montrouzieri - a | 4 | petri dish | 6 | test | 0 | 1 |
| C. montrouzieri - a | 4 | petri dish | 7 | test | 0 | 1 |
| C. montrouzieri - a | 4 | petri dish | 8 | test | 0 | 1 |
| C. montrouzieri - a | 4 | petri dish | 9 | test | 1 | 1 |
| C. montrouzieri - a | 4 | petri dish | 10 | test | 0 | 0 |
| C. montrouzieri - a | 4 | petri dish | 1 | control | 0 | 0 |
| C. montrouzieri - a | 4 | petri dish | 2 | control | 0 | 0 |
| C. montrouzieri - a | 4 | petri dish | 3 | control | 0 | 0 |
| C. montrouzieri - a | 4 | petri dish | 4 | control | 0 | 0 |
| C. montrouzieri - a | 4 | petri dish | 5 | control | 0 | 0 |
| C. montrouzieri - a | 5 | petri dish | 1 | test | 0 | 1 |
| C. montrouzieri - a | 5 | petri dish | 2 | test | 0 | 0 |
| C. montrouzieri - a | 5 | petri dish | 3 | test | 0 | 1 |
| C. montrouzieri - a | 5 | petri dish | 4 | test | 0 | 0 |
| C. montrouzieri - a | 5 | petri dish | 5 | test | 1 | 0 |
| C. montrouzieri - a | 5 | petri dish | 6 | test | 0 | 1 |
| C. montrouzieri - a | 5 | petri dish | 7 | test | 0 | 1 |
| C. montrouzieri - a | 5 | petri dish | 8 | test | 0 | 1 |
| C. montrouzieri - a | 5 | petri dish | 9 | test | 0 | 1 |
| C. montrouzieri - a | 5 | petri dish | 10 | test | 0 | 0 |
| C. montrouzieri - a | 5 | petri dish | 1 | control | 0 | 0 |
| C. montrouzieri - a | 5 | petri dish | 2 | control | 0 | 0 |
| C. montrouzieri - a | 5 | petri dish | 3 | control | 0 | 0 |
| C. montrouzieri - a | 5 | petri dish | 4 | control | 0 | 0 |
| C. montrouzieri - a | 5 | petri dish | 5 | control | 0 | 0 |
| C. montrouzieri - a | a | petri dish | 1 | test | 0 | 0 |
| C. montrouzieri - a | a | petri dish | 2 | test | 0 | 1 |
| C. montrouzieri - a | a | petri dish | 3 | test | 0 | 0 |
| C. montrouzieri - a | a | petri dish | 4 | test | 0 | 0 |
| C. montrouzieri - a | a | petri dish | 5 | test | 0 | 1 |
| C. montrouzieri - a | a | petri dish | 6 | test | 0 | 0 |
| C. montrouzieri - a | a | petri dish | 7 | test | 0 | 0 |
| C. montrouzieri - a | a | petri dish | 8 | test | 0 | 1 |
| C. montrouzieri - a | a | petri dish | 9 | test | 0 | 0 |
| C. montrouzieri - a | a | petri dish | 10 | test | 0 | 0 |
| C. montrouzieri - a | a | petri dish | 1 | control | 0 | 0 |
| C. montrouzieri - a | a | petri dish | 2 | control | 0 | 0 |
| C. montrouzieri - a | a | petri dish | 3 | control | 0 | 0 |
| C. montrouzieri - a | a | petri dish | 4 | control | 0 | 0 |
| C. montrouzieri - a | a | petri dish | 5 | control | 0 | 0 |
| C. montrouzieri - a | 1 | bell bean | 1 | test | 0 | 0 |
| C. montrouzieri - a | 1 | bell bean | 2 | test | 0 | 0 |
| C. montrouzieri - a | 1 | bell bean | 3 | test | 0 | 0 |
| C. montrouzieri - a | 1 | bell bean | 4 | test | 1 | 0 |
| C. montrouzieri - a | 1 | bell bean | 5 | test | 0 | 0 |
| C. montrouzieri - a | 1 | bell bean | 6 | test | 0 | 0 |
| C. montrouzieri - a | 1 | bell bean | 7 | test | 0 | 0 |
| C. montrouzieri - a | 1 | bell bean | 8 | test | 1 | 0 |
| C. montrouzieri - a | 1 | bell bean | 9 | test | 0 | 0 |
| C. montrouzieri - a | 1 | bell bean | 10 | test | 0 | 0 |
| C. montrouzieri - a | 1 | bell bean | 1 | control | 0 | 0 |
| C. montrouzieri - a | 1 | bell bean | 2 | control | 0 | 0 |
| C. montrouzieri - a | 1 | bell bean | 3 | control | 0 | 0 |
| C. montrouzieri - a | 1 | bell bean | 4 | control | 0 | 0 |
| C. montrouzieri - a | 1 | bell bean | 5 | control | 0 | 0 |
| C. montrouzieri - a | 1 | bell bean | 6 | control | 0 | 0 |
| C. montrouzieri - a | 1 | bell bean | 7 | control | 0 | 0 |
| C. montrouzieri - a | 1 | bell bean | 8 | control | 0 | 0 |
| C. montrouzieri - a | 1 | bell bean | 9 | control | 0 | 0 |
| C. montrouzieri - a | 1 | bell bean | 10 | control | 0 | 0 |
| C. montrouzieri - a | 2 | bell bean | 1 | test | 0 | 0 |
| C. montrouzieri - a | 2 | bell bean | 2 | test | 0 | 1 |
| C. montrouzieri - a | 2 | bell bean | 3 | test | 0 | 0 |
| C. montrouzieri - a | 2 | bell bean | 4 | test | 0 | 1 |
| C. montrouzieri - a | 2 | bell bean | 5 | test | 0 | 0 |
| C. montrouzieri - a | 2 | bell bean | 6 | test | 0 | 0 |
| C. montrouzieri - a | 2 | bell bean | 7 | test | 0 | 0 |
| C. montrouzieri - a | 2 | bell bean | 8 | test | 0 | 0 |
| C. montrouzieri - a | 2 | bell bean | 9 | test | 0 | 0 |
| C. montrouzieri - a | 2 | bell bean | 10 | test | 0 | 0 |
| C. montrouzieri - a | 2 | bell bean | 1 | control | 0 | 0 |
| C. montrouzieri - a | 2 | bell bean | 2 | control | 0 | 0 |
| C. montrouzieri - a | 2 | bell bean | 3 | control | 0 | 0 |
| C. montrouzieri - a | 2 | bell bean | 4 | control | 0 | 0 |
| C. montrouzieri - a | 2 | bell bean | 5 | control | 0 | 0 |
| C. montrouzieri - a | 2 | bell bean | 6 | control | 0 | 0 |
| C. montrouzieri - a | 2 | bell bean | 7 | control | 0 | 0 |
| C. montrouzieri - a | 2 | bell bean | 8 | control | 0 | 0 |
| C. montrouzieri - a | 2 | bell bean | 9 | control | 0 | 0 |
| C. montrouzieri - a | 2 | bell bean | 10 | control | 0 | 0 |
| C. montrouzieri - a | 3 | bell bean | 1 | test | 0 | 1 |
| C. montrouzieri - a | 3 | bell bean | 2 | test | 0 | 0 |
| C. montrouzieri - a | 3 | bell bean | 3 | test | 0 | 0 |
| C. montrouzieri - a | 3 | bell bean | 4 | test | 0 | 0 |
| C. montrouzieri - a | 3 | bell bean | 5 | test | 0 | 0 |
| C. montrouzieri - a | 3 | bell bean | 6 | test | 0 | 0 |
| C. montrouzieri - a | 3 | bell bean | 7 | test | 0 | 0 |
| C. montrouzieri - a | 3 | bell bean | 8 | test | 0 | 0 |
| C. montrouzieri - a | 3 | bell bean | 9 | test | 0 | 0 |
| C. montrouzieri - a | 3 | bell bean | 10 | test | 0 | 0 |
| C. montrouzieri - a | 3 | bell bean | 1 | control | 0 | 0 |
| C. montrouzieri - a | 3 | bell bean | 2 | control | 0 | 0 |
| C. montrouzieri - a | 3 | bell bean | 3 | control | 0 | 0 |
| C. montrouzieri - a | 3 | bell bean | 4 | control | 0 | 0 |
| C. montrouzieri - a | 3 | bell bean | 5 | control | 0 | 0 |
| C. montrouzieri - a | 3 | bell bean | 6 | control | 0 | 0 |
| C. montrouzieri - a | 3 | bell bean | 7 | control | 0 | 0 |
| C. montrouzieri - a | 3 | bell bean | 8 | control | 0 | 0 |
| C. montrouzieri - a | 3 | bell bean | 9 | control | 0 | 0 |
| C. montrouzieri - a | 3 | bell bean | 10 | control | 0 | 0 |
| C. montrouzieri - a | 4 | bell bean | 1 | test | 0 | 0 |
| C. montrouzieri - a | 4 | bell bean | 2 | test | 0 | 0 |
| C. montrouzieri - a | 4 | bell bean | 3 | test | 1 | 0 |
| C. montrouzieri - a | 4 | bell bean | 4 | test | 0 | 0 |
| C. montrouzieri - a | 4 | bell bean | 5 | test | 0 | 0 |
| C. montrouzieri - a | 4 | bell bean | 6 | test | 0 | 0 |
| C. montrouzieri - a | 4 | bell bean | 7 | test | 0 | 0 |
| C. montrouzieri - a | 4 | bell bean | 8 | test | 0 | 1 |
| C. montrouzieri - a | 4 | bell bean | 9 | test | 0 | 0 |
| C. montrouzieri - a | 4 | bell bean | 10 | test | 0 | 0 |
| C. montrouzieri - a | 4 | bell bean | 1 | control | 0 | 0 |
| C. montrouzieri - a | 4 | bell bean | 2 | control | 0 | 0 |
| C. montrouzieri - a | 4 | bell bean | 3 | control | 0 | 0 |
| C. montrouzieri - a | 4 | bell bean | 4 | control | 0 | 0 |
| C. montrouzieri - a | 4 | bell bean | 5 | control | 0 | 0 |
| C. montrouzieri - a | 4 | bell bean | 6 | control | 1 | 0 |
| C. montrouzieri - a | 4 | bell bean | 7 | control | 0 | 0 |
| C. montrouzieri - a | 4 | bell bean | 8 | control | 0 | 0 |
| C. montrouzieri - a | 4 | bell bean | 9 | control | 0 | 0 |
| C. montrouzieri - a | 4 | bell bean | 10 | control | 0 | 0 |
| C. montrouzieri - a | 5 | bell bean | 1 | test | 0 | 0 |
| C. montrouzieri - a | 5 | bell bean | 2 | test | 0 | 0 |
| C. montrouzieri - a | 5 | bell bean | 3 | test | 0 | 0 |
| C. montrouzieri - a | 5 | bell bean | 4 | test | 0 | 0 |
| C. montrouzieri - a | 5 | bell bean | 5 | test | 0 | 0 |
| C. montrouzieri - a | 5 | bell bean | 6 | test | 0 | 1 |
| C. montrouzieri - a | 5 | bell bean | 7 | test | 0 | 0 |
| C. montrouzieri - a | 5 | bell bean | 8 | test | 0 | 0 |
| C. montrouzieri - a | 5 | bell bean | 9 | test | 0 | 0 |
| C. montrouzieri - a | 5 | bell bean | 10 | test | 0 | 0 |
| C. montrouzieri - a | 5 | bell bean | 1 | control | 0 | 0 |
| C. montrouzieri - a | 5 | bell bean | 2 | control | 0 | 0 |
| C. montrouzieri - a | 5 | bell bean | 3 | control | 0 | 0 |
| C. montrouzieri - a | 5 | bell bean | 4 | control | 0 | 0 |
| C. montrouzieri - a | 5 | bell bean | 5 | control | 0 | 0 |
| C. montrouzieri - a | 5 | bell bean | 6 | control | 0 | 0 |
| C. montrouzieri - a | 5 | bell bean | 7 | control | 0 | 0 |
| C. montrouzieri - a | 5 | bell bean | 8 | control | 0 | 0 |
| C. montrouzieri - a | 5 | bell bean | 9 | control | 0 | 0 |
| C. montrouzieri - a | 5 | bell bean | 10 | control | 0 | 0 |
| C. montrouzieri - a | a | bell bean | 1 | test | 0 | 0 |
| C. montrouzieri - a | a | bell bean | 2 | test | 0 | 0 |
| C. montrouzieri - a | a | bell bean | 3 | test | 0 | 0 |
| C. montrouzieri - a | a | bell bean | 4 | test | 0 | 0 |
| C. montrouzieri - a | a | bell bean | 5 | test | 1 | 0 |
| C. montrouzieri - a | a | bell bean | 6 | test | 0 | 0 |
| C. montrouzieri - a | a | bell bean | 7 | test | 0 | 0 |
| C. montrouzieri - a | a | bell bean | 8 | test | 0 | 0 |
| C. montrouzieri - a | a | bell bean | 9 | test | 0 | 0 |
| C. montrouzieri - a | a | bell bean | 10 | test | 1 | 0 |
| C. montrouzieri - a | a | bell bean | 1 | control | 0 | 0 |
| C. montrouzieri - a | a | bell bean | 2 | control | 0 | 0 |
| C. montrouzieri - a | a | bell bean | 3 | control | 0 | 0 |
| C. montrouzieri - a | a | bell bean | 4 | control | 0 | 0 |
| C. montrouzieri - a | a | bell bean | 5 | control | 1 | 0 |
| C. montrouzieri - a | a | bell bean | 6 | control | 0 | 0 |
| C. montrouzieri - a | a | bell bean | 7 | control | 0 | 0 |
| C. montrouzieri - a | a | bell bean | 8 | control | 0 | 0 |
| C. montrouzieri - a | a | bell bean | 9 | control | 0 | 0 |
| C. montrouzieri - a | a | bell bean | 10 | control | 0 | 0 |
| C. rufilabris - l | 1 | petri dish | 1 | test | 1 | 0 |
| C. rufilabris - l | 1 | petri dish | 2 | test | 1 | 0 |
| C. rufilabris - l | 1 | petri dish | 3 | test | 1 | 0 |
| C. rufilabris - l | 1 | petri dish | 4 | test | 1 | 0 |
| C. rufilabris - l | 1 | petri dish | 5 | test | 1 | 0 |
| C. rufilabris - l | 1 | petri dish | 6 | test | 1 | 0 |
| C. rufilabris - l | 1 | petri dish | 7 | test | 1 | 0 |
| C. rufilabris - l | 1 | petri dish | 8 | test | 1 | 0 |
| C. rufilabris - l | 1 | petri dish | 9 | test | 1 | 0 |
| C. rufilabris - l | 1 | petri dish | 10 | test | 1 | 0 |
| C. rufilabris - l | 1 | petri dish | 1 | control | 0 | 0 |
| C. rufilabris - l | 1 | petri dish | 2 | control | 0 | 0 |
| C. rufilabris - l | 1 | petri dish | 3 | control | 0 | 0 |
| C. rufilabris - l | 1 | petri dish | 4 | control | 0 | 0 |
| C. rufilabris - l | 1 | petri dish | 5 | control | 0 | 0 |
| C. rufilabris - l | 2 | petri dish | 1 | test | 1 | 0 |
| C. rufilabris - l | 2 | petri dish | 2 | test | 1 | 0 |
| C. rufilabris - l | 2 | petri dish | 3 | test | 1 | 0 |
| C. rufilabris - l | 2 | petri dish | 4 | test | 1 | 0 |
| C. rufilabris - l | 2 | petri dish | 5 | test | 1 | 0 |
| C. rufilabris - l | 2 | petri dish | 6 | test | 1 | 0 |
| C. rufilabris - l | 2 | petri dish | 7 | test | 1 | 0 |
| C. rufilabris - l | 2 | petri dish | 8 | test | 1 | 0 |
| C. rufilabris - l | 2 | petri dish | 9 | test | 1 | 0 |
| C. rufilabris - l | 2 | petri dish | 10 | test | 1 | 0 |
| C. rufilabris - l | 2 | petri dish | 1 | control | 0 | 0 |
| C. rufilabris - l | 2 | petri dish | 2 | control | 0 | 0 |
| C. rufilabris - l | 2 | petri dish | 3 | control | 0 | 0 |
| C. rufilabris - l | 2 | petri dish | 4 | control | 0 | 0 |
| C. rufilabris - l | 2 | petri dish | 5 | control | 0 | 0 |
| C. rufilabris - l | 3 | petri dish | 1 | test | 1 | 0 |
| C. rufilabris - l | 3 | petri dish | 2 | test | 1 | 0 |
| C. rufilabris - l | 3 | petri dish | 3 | test | 1 | 0 |
| C. rufilabris - l | 3 | petri dish | 4 | test | 1 | 0 |
| C. rufilabris - l | 3 | petri dish | 5 | test | 1 | 0 |
| C. rufilabris - l | 3 | petri dish | 6 | test | 1 | 0 |
| C. rufilabris - l | 3 | petri dish | 7 | test | 1 | 0 |
| C. rufilabris - l | 3 | petri dish | 8 | test | 1 | 0 |
| C. rufilabris - l | 3 | petri dish | 9 | test | 1 | 0 |
| C. rufilabris - l | 3 | petri dish | 10 | test | 1 | 0 |
| C. rufilabris - l | 3 | petri dish | 1 | control | 0 | 0 |
| C. rufilabris - l | 3 | petri dish | 2 | control | 0 | 0 |
| C. rufilabris - l | 3 | petri dish | 3 | control | 0 | 0 |
| C. rufilabris - l | 3 | petri dish | 4 | control | 0 | 0 |
| C. rufilabris - l | 3 | petri dish | 5 | control | 0 | 0 |
| C. rufilabris - l | 4 | petri dish | 1 | test | 1 | 0 |
| C. rufilabris - l | 4 | petri dish | 2 | test | 1 | 0 |
| C. rufilabris - l | 4 | petri dish | 3 | test | 1 | 0 |
| C. rufilabris - l | 4 | petri dish | 4 | test | 1 | 0 |
| C. rufilabris - l | 4 | petri dish | 5 | test | 1 | 0 |
| C. rufilabris - l | 4 | petri dish | 6 | test | 1 | 0 |
| C. rufilabris - l | 4 | petri dish | 7 | test | 0 | 0 |
| C. rufilabris - l | 4 | petri dish | 8 | test | 1 | 0 |
| C. rufilabris - l | 4 | petri dish | 9 | test | 1 | 0 |
| C. rufilabris - l | 4 | petri dish | 10 | test | 1 | 0 |
| C. rufilabris - l | 4 | petri dish | 1 | control | 0 | 0 |
| C. rufilabris - l | 4 | petri dish | 2 | control | 0 | 0 |
| C. rufilabris - l | 4 | petri dish | 3 | control | 0 | 0 |
| C. rufilabris - l | 4 | petri dish | 4 | control | 0 | 0 |
| C. rufilabris - l | 4 | petri dish | 5 | control | 0 | 0 |
| C. rufilabris - l | 5 | petri dish | 1 | test | 1 | 0 |
| C. rufilabris - l | 5 | petri dish | 2 | test | 1 | 0 |
| C. rufilabris - l | 5 | petri dish | 3 | test | 1 | 0 |
| C. rufilabris - l | 5 | petri dish | 4 | test | 1 | 0 |
| C. rufilabris - l | 5 | petri dish | 5 | test | 1 | 0 |
| C. rufilabris - l | 5 | petri dish | 6 | test | 1 | 0 |
| C. rufilabris - l | 5 | petri dish | 7 | test | 1 | 0 |
| C. rufilabris - l | 5 | petri dish | 8 | test | 1 | 0 |
| C. rufilabris - l | 5 | petri dish | 9 | test | 1 | 0 |
| C. rufilabris - l | 5 | petri dish | 10 | test | 1 | 0 |
| C. rufilabris - l | 5 | petri dish | 1 | control | 0 | 0 |
| C. rufilabris - l | 5 | petri dish | 2 | control | 0 | 0 |
| C. rufilabris - l | 5 | petri dish | 3 | control | 0 | 0 |
| C. rufilabris - l | 5 | petri dish | 4 | control | 0 | 0 |
| C. rufilabris - l | 5 | petri dish | 5 | control | 0 | 0 |
| C. rufilabris - l | a | petri dish | 1 | test | 0 | 0 |
| C. rufilabris - l | a | petri dish | 2 | test | 0 | 0 |
| C. rufilabris - l | a | petri dish | 3 | test | 0 | 0 |
| C. rufilabris - l | a | petri dish | 4 | test | 0 | 0 |
| C. rufilabris - l | a | petri dish | 5 | test | 0 | 0 |
| C. rufilabris - l | a | petri dish | 6 | test | 0 | 0 |
| C. rufilabris - l | a | petri dish | 7 | test | 0 | 0 |
| C. rufilabris - l | a | petri dish | 8 | test | 0 | 0 |
| C. rufilabris - l | a | petri dish | 9 | test | 0 | 0 |
| C. rufilabris - l | a | petri dish | 10 | test | 0 | 0 |
| C. rufilabris - l | a | petri dish | 1 | control | 0 | 0 |
| C. rufilabris - l | a | petri dish | 2 | control | 0 | 0 |
| C. rufilabris - l | a | petri dish | 3 | control | 0 | 0 |
| C. rufilabris - l | a | petri dish | 4 | control | 0 | 0 |
| C. rufilabris - l | a | petri dish | 5 | control | 0 | 0 |
| C. rufilabris - l | 1 | bell bean | 1 | test | 0 | 0 |
| C. rufilabris - l | 1 | bell bean | 2 | test | 1 | 0 |
| C. rufilabris - l | 1 | bell bean | 3 | test | 0 | 0 |
| C. rufilabris - l | 1 | bell bean | 4 | test | 1 | 0 |
| C. rufilabris - l | 1 | bell bean | 5 | test | 1 | 0 |
| C. rufilabris - l | 1 | bell bean | 6 | test | 0 | 0 |
| C. rufilabris - l | 1 | bell bean | 7 | test | 1 | 0 |
| C. rufilabris - l | 1 | bell bean | 8 | test | 1 | 0 |
| C. rufilabris - l | 1 | bell bean | 9 | test | 1 | 0 |
| C. rufilabris - l | 1 | bell bean | 10 | test | 1 | 0 |
| C. rufilabris - l | 1 | bell bean | 1 | control | 0 | 0 |
| C. rufilabris - l | 1 | bell bean | 2 | control | 0 | 0 |
| C. rufilabris - l | 1 | bell bean | 3 | control | 0 | 0 |
| C. rufilabris - l | 1 | bell bean | 4 | control | 0 | 0 |
| C. rufilabris - l | 1 | bell bean | 5 | control | 0 | 0 |
| C. rufilabris - l | 1 | bell bean | 6 | control | 0 | 0 |
| C. rufilabris - l | 1 | bell bean | 7 | control | 1 | 0 |
| C. rufilabris - l | 1 | bell bean | 8 | control | 0 | 0 |
| C. rufilabris - l | 1 | bell bean | 9 | control | 0 | 0 |
| C. rufilabris - l | 1 | bell bean | 10 | control | 0 | 0 |
| C. rufilabris - l | 2 | bell bean | 1 | test | 1 | 0 |
| C. rufilabris - l | 2 | bell bean | 2 | test | 1 | 0 |
| C. rufilabris - l | 2 | bell bean | 3 | test | 1 | 0 |
| C. rufilabris - l | 2 | bell bean | 4 | test | 1 | 0 |
| C. rufilabris - l | 2 | bell bean | 5 | test | 1 | 0 |
| C. rufilabris - l | 2 | bell bean | 6 | test | 0 | 0 |
| C. rufilabris - l | 2 | bell bean | 7 | test | 1 | 0 |
| C. rufilabris - l | 2 | bell bean | 8 | test | 0 | 0 |
| C. rufilabris - l | 2 | bell bean | 9 | test | 0 | 0 |
| C. rufilabris - l | 2 | bell bean | 10 | test | 1 | 0 |
| C. rufilabris - l | 2 | bell bean | 1 | control | 0 | 0 |
| C. rufilabris - l | 2 | bell bean | 2 | control | 0 | 0 |
| C. rufilabris - l | 2 | bell bean | 3 | control | 0 | 0 |
| C. rufilabris - l | 2 | bell bean | 4 | control | 0 | 0 |
| C. rufilabris - l | 2 | bell bean | 5 | control | 0 | 0 |
| C. rufilabris - l | 2 | bell bean | 6 | control | 0 | 0 |
| C. rufilabris - l | 2 | bell bean | 7 | control | 0 | 0 |
| C. rufilabris - l | 2 | bell bean | 8 | control | 0 | 0 |
| C. rufilabris - l | 2 | bell bean | 9 | control | 0 | 0 |
| C. rufilabris - l | 2 | bell bean | 10 | control | 1 | 0 |
| C. rufilabris - l | 3 | bell bean | 1 | test | 1 | 0 |
| C. rufilabris - l | 3 | bell bean | 2 | test | 0 | 0 |
| C. rufilabris - l | 3 | bell bean | 3 | test | 0 | 0 |
| C. rufilabris - l | 3 | bell bean | 4 | test | 0 | 0 |
| C. rufilabris - l | 3 | bell bean | 5 | test | 0 | 0 |
| C. rufilabris - l | 3 | bell bean | 6 | test | 1 | 0 |
| C. rufilabris - l | 3 | bell bean | 7 | test | 0 | 0 |
| C. rufilabris - l | 3 | bell bean | 8 | test | 0 | 0 |
| C. rufilabris - l | 3 | bell bean | 9 | test | 0 | 0 |
| C. rufilabris - l | 3 | bell bean | 10 | test | 1 | 0 |
| C. rufilabris - l | 3 | bell bean | 1 | control | 0 | 0 |
| C. rufilabris - l | 3 | bell bean | 2 | control | 0 | 0 |
| C. rufilabris - l | 3 | bell bean | 3 | control | 0 | 0 |
| C. rufilabris - l | 3 | bell bean | 4 | control | 0 | 0 |
| C. rufilabris - l | 3 | bell bean | 5 | control | 0 | 0 |
| C. rufilabris - l | 3 | bell bean | 6 | control | 0 | 0 |
| C. rufilabris - l | 3 | bell bean | 7 | control | 0 | 0 |
| C. rufilabris - l | 3 | bell bean | 8 | control | 0 | 0 |
| C. rufilabris - l | 3 | bell bean | 9 | control | 0 | 0 |
| C. rufilabris - l | 3 | bell bean | 10 | control | 0 | 0 |
| C. rufilabris - l | 4 | bell bean | 1 | test | 1 | 0 |
| C. rufilabris - l | 4 | bell bean | 2 | test | 0 | 0 |
| C. rufilabris - l | 4 | bell bean | 3 | test | 1 | 0 |
| C. rufilabris - l | 4 | bell bean | 4 | test | 1 | 0 |
| C. rufilabris - l | 4 | bell bean | 5 | test | 0 | 0 |
| C. rufilabris - l | 4 | bell bean | 6 | test | 0 | 0 |
| C. rufilabris - l | 4 | bell bean | 7 | test | 1 | 0 |
| C. rufilabris - l | 4 | bell bean | 8 | test | 0 | 0 |
| C. rufilabris - l | 4 | bell bean | 9 | test | 1 | 0 |
| C. rufilabris - l | 4 | bell bean | 10 | test | 0 | 0 |
| C. rufilabris - l | 4 | bell bean | 1 | control | 0 | 0 |
| C. rufilabris - l | 4 | bell bean | 2 | control | 0 | 0 |
| C. rufilabris - l | 4 | bell bean | 3 | control | 0 | 0 |
| C. rufilabris - l | 4 | bell bean | 4 | control | 0 | 0 |
| C. rufilabris - l | 4 | bell bean | 5 | control | 0 | 0 |
| C. rufilabris - l | 4 | bell bean | 6 | control | 0 | 0 |
| C. rufilabris - l | 4 | bell bean | 7 | control | 0 | 0 |
| C. rufilabris - l | 4 | bell bean | 8 | control | 0 | 0 |
| C. rufilabris - l | 4 | bell bean | 9 | control | 0 | 0 |
| C. rufilabris - l | 4 | bell bean | 10 | control | 0 | 0 |
| C. rufilabris - l | 5 | bell bean | 1 | test | 1 | 0 |
| C. rufilabris - l | 5 | bell bean | 2 | test | 1 | 0 |
| C. rufilabris - l | 5 | bell bean | 3 | test | 0 | 0 |
| C. rufilabris - l | 5 | bell bean | 4 | test | 1 | 0 |
| C. rufilabris - l | 5 | bell bean | 5 | test | 0 | 0 |
| C. rufilabris - l | 5 | bell bean | 6 | test | 0 | 0 |
| C. rufilabris - l | 5 | bell bean | 7 | test | 0 | 0 |
| C. rufilabris - l | 5 | bell bean | 8 | test | 0 | 0 |
| C. rufilabris - l | 5 | bell bean | 9 | test | 1 | 0 |
| C. rufilabris - l | 5 | bell bean | 10 | test | 1 | 0 |
| C. rufilabris - l | 5 | bell bean | 1 | control | 0 | 0 |
| C. rufilabris - l | 5 | bell bean | 2 | control | 0 | 0 |
| C. rufilabris - l | 5 | bell bean | 3 | control | 0 | 0 |
| C. rufilabris - l | 5 | bell bean | 4 | control | 0 | 0 |
| C. rufilabris - l | 5 | bell bean | 5 | control | 0 | 0 |
| C. rufilabris - l | 5 | bell bean | 6 | control | 0 | 0 |
| C. rufilabris - l | 5 | bell bean | 7 | control | 0 | 0 |
| C. rufilabris - l | 5 | bell bean | 8 | control | 0 | 0 |
| C. rufilabris - l | 5 | bell bean | 9 | control | 0 | 0 |
| C. rufilabris - l | 5 | bell bean | 10 | control | 0 | 0 |
| C. rufilabris - l | a | bell bean | 1 | test | 0 | 0 |
| C. rufilabris - l | a | bell bean | 2 | test | 0 | 0 |
| C. rufilabris - l | a | bell bean | 3 | test | 0 | 0 |
| C. rufilabris - l | a | bell bean | 4 | test | 0 | 0 |
| C. rufilabris - l | a | bell bean | 5 | test | 0 | 0 |
| C. rufilabris - l | a | bell bean | 6 | test | 0 | 0 |
| C. rufilabris - l | a | bell bean | 7 | test | 0 | 0 |
| C. rufilabris - l | a | bell bean | 8 | test | 0 | 0 |
| C. rufilabris - l | a | bell bean | 9 | test | 0 | 0 |
| C. rufilabris - l | a | bell bean | 10 | test | 0 | 0 |
| C. rufilabris - l | a | bell bean | 1 | control | 0 | 0 |
| C. rufilabris - l | a | bell bean | 2 | control | 0 | 0 |
| C. rufilabris - l | a | bell bean | 3 | control | 0 | 0 |
| C. rufilabris - l | a | bell bean | 4 | control | 0 | 0 |
| C. rufilabris - l | a | bell bean | 5 | control | 0 | 0 |
| C. rufilabris - l | a | bell bean | 6 | control | 0 | 0 |
| C. rufilabris - l | a | bell bean | 7 | control | 0 | 0 |
| C. rufilabris - l | a | bell bean | 8 | control | 0 | 0 |
| C. rufilabris - l | a | bell bean | 9 | control | 0 | 0 |
| C. rufilabris - l | a | bell bean | 10 | control | 0 | 0 |
| S. barberi - a | 1 | petri dish | 1 | test | 0 | 0 |
| S. barberi - a | 1 | petri dish | 2 | test | 0 | 0 |
| S. barberi - a | 1 | petri dish | 3 | test | 0 | 0 |
| S. barberi - a | 1 | petri dish | 4 | test | 0 | 0 |
| S. barberi - a | 1 | petri dish | 5 | test | 0 | 0 |
| S. barberi - a | 1 | petri dish | 6 | test | 0 | 0 |
| S. barberi - a | 1 | petri dish | 7 | test | 0 | 0 |
| S. barberi - a | 1 | petri dish | 8 | test | 0 | 0 |
| S. barberi - a | 1 | petri dish | 9 | test | 0 | 0 |
| S. barberi - a | 1 | petri dish | 10 | test | 0 | 1 |
| S. barberi - a | 1 | petri dish | 1 | control | 0 | 0 |
| S. barberi - a | 1 | petri dish | 2 | control | 0 | 0 |
| S. barberi - a | 1 | petri dish | 3 | control | 0 | 0 |
| S. barberi - a | 1 | petri dish | 4 | control | 0 | 0 |
| S. barberi - a | 1 | petri dish | 5 | control | 0 | 0 |
| S. barberi - a | 2 | petri dish | 1 | test | 0 | 0 |
| S. barberi - a | 2 | petri dish | 2 | test | 0 | 0 |
| S. barberi - a | 2 | petri dish | 3 | test | 0 | 1 |
| S. barberi - a | 2 | petri dish | 4 | test | 0 | 0 |
| S. barberi - a | 2 | petri dish | 5 | test | 0 | 0 |
| S. barberi - a | 2 | petri dish | 6 | test | 0 | 0 |
| S. barberi - a | 2 | petri dish | 7 | test | 0 | 0 |
| S. barberi - a | 2 | petri dish | 8 | test | 0 | 0 |
| S. barberi - a | 2 | petri dish | 9 | test | 0 | 0 |
| S. barberi - a | 2 | petri dish | 10 | test | 0 | 0 |
| S. barberi - a | 2 | petri dish | 1 | control | 0 | 0 |
| S. barberi - a | 2 | petri dish | 2 | control | 0 | 0 |
| S. barberi - a | 2 | petri dish | 3 | control | 0 | 0 |
| S. barberi - a | 2 | petri dish | 4 | control | 0 | 0 |
| S. barberi - a | 2 | petri dish | 5 | control | 0 | 1 |
| S. barberi - a | 3 | petri dish | 1 | test | 0 | 1 |
| S. barberi - a | 3 | petri dish | 2 | test | 0 | 0 |
| S. barberi - a | 3 | petri dish | 3 | test | 0 | 0 |
| S. barberi - a | 3 | petri dish | 4 | test | 0 | 0 |
| S. barberi - a | 3 | petri dish | 5 | test | 0 | 0 |
| S. barberi - a | 3 | petri dish | 6 | test | 0 | 0 |
| S. barberi - a | 3 | petri dish | 7 | test | 0 | 0 |
| S. barberi - a | 3 | petri dish | 8 | test | 0 | 0 |
| S. barberi - a | 3 | petri dish | 9 | test | 0 | 1 |
| S. barberi - a | 3 | petri dish | 10 | test | 0 | 0 |
| S. barberi - a | 3 | petri dish | 1 | control | 0 | 0 |
| S. barberi - a | 3 | petri dish | 2 | control | 0 | 0 |
| S. barberi - a | 3 | petri dish | 3 | control | 0 | 0 |
| S. barberi - a | 3 | petri dish | 4 | control | 0 | 0 |
| S. barberi - a | 3 | petri dish | 5 | control | 0 | 0 |
| S. barberi - a | 4 | petri dish | 1 | test | 0 | 0 |
| S. barberi - a | 4 | petri dish | 2 | test | 0 | 0 |
| S. barberi - a | 4 | petri dish | 3 | test | 0 | 0 |
| S. barberi - a | 4 | petri dish | 4 | test | 1 | 0 |
| S. barberi - a | 4 | petri dish | 5 | test | 0 | 0 |
| S. barberi - a | 4 | petri dish | 6 | test | 1 | 1 |
| S. barberi - a | 4 | petri dish | 7 | test | 0 | 0 |
| S. barberi - a | 4 | petri dish | 8 | test | 0 | 0 |
| S. barberi - a | 4 | petri dish | 9 | test | 0 | 0 |
| S. barberi - a | 4 | petri dish | 10 | test | 0 | 0 |
| S. barberi - a | 4 | petri dish | 1 | control | 0 | 0 |
| S. barberi - a | 4 | petri dish | 2 | control | 0 | 0 |
| S. barberi - a | 4 | petri dish | 3 | control | 0 | 0 |
| S. barberi - a | 4 | petri dish | 4 | control | 0 | 0 |
| S. barberi - a | 4 | petri dish | 5 | control | 0 | 0 |
| S. barberi - a | 5 | petri dish | 1 | test | 0 | 0 |
| S. barberi - a | 5 | petri dish | 2 | test | 0 | 0 |
| S. barberi - a | 5 | petri dish | 3 | test | 1 | 1 |
| S. barberi - a | 5 | petri dish | 4 | test | 0 | 1 |
| S. barberi - a | 5 | petri dish | 5 | test | 0 | 0 |
| S. barberi - a | 5 | petri dish | 6 | test | 0 | 0 |
| S. barberi - a | 5 | petri dish | 7 | test | 0 | 0 |
| S. barberi - a | 5 | petri dish | 8 | test | 0 | 0 |
| S. barberi - a | 5 | petri dish | 9 | test | 0 | 0 |
| S. barberi - a | 5 | petri dish | 10 | test | 0 | 0 |
| S. barberi - a | 5 | petri dish | 1 | control | 0 | 0 |
| S. barberi - a | 5 | petri dish | 2 | control | 0 | 0 |
| S. barberi - a | 5 | petri dish | 3 | control | 0 | 0 |
| S. barberi - a | 5 | petri dish | 4 | control | 0 | 0 |
| S. barberi - a | 5 | petri dish | 5 | control | 0 | 0 |
| S. barberi - a | a | petri dish | 1 | test | 0 | 0 |
| S. barberi - a | a | petri dish | 2 | test | 1 | 0 |
| S. barberi - a | a | petri dish | 3 | test | 1 | 1 |
| S. barberi - a | a | petri dish | 4 | test | 1 | 0 |
| S. barberi - a | a | petri dish | 5 | test | 1 | 0 |
| S. barberi - a | a | petri dish | 6 | test | 0 | 0 |
| S. barberi - a | a | petri dish | 7 | test | 0 | 0 |
| S. barberi - a | a | petri dish | 8 | test | 0 | 0 |
| S. barberi - a | a | petri dish | 9 | test | 0 | 0 |
| S. barberi - a | a | petri dish | 10 | test | 0 | 1 |
| S. barberi - a | a | petri dish | 1 | control | 1 | 0 |
| S. barberi - a | a | petri dish | 2 | control | 0 | 0 |
| S. barberi - a | a | petri dish | 3 | control | 1 | 0 |
| S. barberi - a | a | petri dish | 4 | control | 0 | 0 |
| S. barberi - a | a | petri dish | 5 | control | 0 | 0 |
| S. barberi - a | 1 | bell bean | 1 | test | 0 | 0 |
| S. barberi - a | 1 | bell bean | 2 | test | 0 | 0 |
| S. barberi - a | 1 | bell bean | 3 | test | 0 | 0 |
| S. barberi - a | 1 | bell bean | 4 | test | 0 | 0 |
| S. barberi - a | 1 | bell bean | 5 | test | 0 | 0 |
| S. barberi - a | 1 | bell bean | 6 | test | 0 | 0 |
| S. barberi - a | 1 | bell bean | 7 | test | 0 | 0 |
| S. barberi - a | 1 | bell bean | 8 | test | 0 | 0 |
| S. barberi - a | 1 | bell bean | 9 | test | 0 | 0 |
| S. barberi - a | 1 | bell bean | 10 | test | 0 | 0 |
| S. barberi - a | 1 | bell bean | 1 | control | 0 | 0 |
| S. barberi - a | 1 | bell bean | 2 | control | 0 | 0 |
| S. barberi - a | 1 | bell bean | 3 | control | 0 | 0 |
| S. barberi - a | 1 | bell bean | 4 | control | 1 | 0 |
| S. barberi - a | 1 | bell bean | 5 | control | 0 | 0 |
| S. barberi - a | 1 | bell bean | 6 | control | 0 | 0 |
| S. barberi - a | 1 | bell bean | 7 | control | 0 | 0 |
| S. barberi - a | 1 | bell bean | 8 | control | 0 | 0 |
| S. barberi - a | 1 | bell bean | 9 | control | 0 | 0 |
| S. barberi - a | 1 | bell bean | 10 | control | 0 | 0 |
| S. barberi - a | 2 | bell bean | 1 | test | 0 | 0 |
| S. barberi - a | 2 | bell bean | 2 | test | 0 | 0 |
| S. barberi - a | 2 | bell bean | 3 | test | 0 | 0 |
| S. barberi - a | 2 | bell bean | 4 | test | 0 | 0 |
| S. barberi - a | 2 | bell bean | 5 | test | 0 | 0 |
| S. barberi - a | 2 | bell bean | 6 | test | 0 | 1 |
| S. barberi - a | 2 | bell bean | 7 | test | 0 | 0 |
| S. barberi - a | 2 | bell bean | 8 | test | 0 | 0 |
| S. barberi - a | 2 | bell bean | 9 | test | 0 | 0 |
| S. barberi - a | 2 | bell bean | 10 | test | 0 | 0 |
| S. barberi - a | 2 | bell bean | 1 | control | 0 | 0 |
| S. barberi - a | 2 | bell bean | 2 | control | 0 | 0 |
| S. barberi - a | 2 | bell bean | 3 | control | 0 | 0 |
| S. barberi - a | 2 | bell bean | 4 | control | 0 | 0 |
| S. barberi - a | 2 | bell bean | 5 | control | 0 | 0 |
| S. barberi - a | 2 | bell bean | 6 | control | 0 | 0 |
| S. barberi - a | 2 | bell bean | 7 | control | 0 | 0 |
| S. barberi - a | 2 | bell bean | 8 | control | 0 | 0 |
| S. barberi - a | 2 | bell bean | 9 | control | 0 | 0 |
| S. barberi - a | 2 | bell bean | 10 | control | 0 | 0 |
| S. barberi - a | 3 | bell bean | 1 | test | 0 | 0 |
| S. barberi - a | 3 | bell bean | 2 | test | 0 | 0 |
| S. barberi - a | 3 | bell bean | 3 | test | 0 | 0 |
| S. barberi - a | 3 | bell bean | 4 | test | 0 | 0 |
| S. barberi - a | 3 | bell bean | 5 | test | 0 | 0 |
| S. barberi - a | 3 | bell bean | 6 | test | 0 | 0 |
| S. barberi - a | 3 | bell bean | 7 | test | 0 | 0 |
| S. barberi - a | 3 | bell bean | 8 | test | 0 | 0 |
| S. barberi - a | 3 | bell bean | 9 | test | 0 | 0 |
| S. barberi - a | 3 | bell bean | 10 | test | 0 | 0 |
| S. barberi - a | 3 | bell bean | 1 | control | 0 | 0 |
| S. barberi - a | 3 | bell bean | 2 | control | 0 | 0 |
| S. barberi - a | 3 | bell bean | 3 | control | 0 | 0 |
| S. barberi - a | 3 | bell bean | 4 | control | 0 | 0 |
| S. barberi - a | 3 | bell bean | 5 | control | 0 | 0 |
| S. barberi - a | 3 | bell bean | 6 | control | 0 | 0 |
| S. barberi - a | 3 | bell bean | 7 | control | 0 | 0 |
| S. barberi - a | 3 | bell bean | 8 | control | 0 | 0 |
| S. barberi - a | 3 | bell bean | 9 | control | 0 | 0 |
| S. barberi - a | 3 | bell bean | 10 | control | 0 | 0 |
| S. barberi - a | 4 | bell bean | 1 | test | 0 | 0 |
| S. barberi - a | 4 | bell bean | 2 | test | 0 | 0 |
| S. barberi - a | 4 | bell bean | 3 | test | 0 | 0 |
| S. barberi - a | 4 | bell bean | 4 | test | 0 | 0 |
| S. barberi - a | 4 | bell bean | 5 | test | 0 | 0 |
| S. barberi - a | 4 | bell bean | 6 | test | 0 | 0 |
| S. barberi - a | 4 | bell bean | 7 | test | 0 | 0 |
| S. barberi - a | 4 | bell bean | 8 | test | 0 | 0 |
| S. barberi - a | 4 | bell bean | 9 | test | 0 | 0 |
| S. barberi - a | 4 | bell bean | 10 | test | 0 | 0 |
| S. barberi - a | 4 | bell bean | 1 | control | 0 | 0 |
| S. barberi - a | 4 | bell bean | 2 | control | 0 | 0 |
| S. barberi - a | 4 | bell bean | 3 | control | 1 | 0 |
| S. barberi - a | 4 | bell bean | 4 | control | 0 | 0 |
| S. barberi - a | 4 | bell bean | 5 | control | 0 | 0 |
| S. barberi - a | 4 | bell bean | 6 | control | 0 | 0 |
| S. barberi - a | 4 | bell bean | 7 | control | 0 | 0 |
| S. barberi - a | 4 | bell bean | 8 | control | 0 | 0 |
| S. barberi - a | 4 | bell bean | 9 | control | 1 | 0 |
| S. barberi - a | 4 | bell bean | 10 | control | 0 | 0 |
| S. barberi - a | 5 | bell bean | 1 | test | 0 | 0 |
| S. barberi - a | 5 | bell bean | 2 | test | 0 | 0 |
| S. barberi - a | 5 | bell bean | 3 | test | 0 | 0 |
| S. barberi - a | 5 | bell bean | 4 | test | 0 | 0 |
| S. barberi - a | 5 | bell bean | 5 | test | 0 | 1 |
| S. barberi - a | 5 | bell bean | 6 | test | 0 | 0 |
| S. barberi - a | 5 | bell bean | 7 | test | 0 | 1 |
| S. barberi - a | 5 | bell bean | 8 | test | 0 | 0 |
| S. barberi - a | 5 | bell bean | 9 | test | 0 | 0 |
| S. barberi - a | 5 | bell bean | 10 | test | 0 | 1 |
| S. barberi - a | 5 | bell bean | 1 | control | 0 | 0 |
| S. barberi - a | 5 | bell bean | 2 | control | 1 | 0 |
| S. barberi - a | 5 | bell bean | 3 | control | 1 | 0 |
| S. barberi - a | 5 | bell bean | 4 | control | 0 | 0 |
| S. barberi - a | 5 | bell bean | 5 | control | 0 | 0 |
| S. barberi - a | 5 | bell bean | 6 | control | 0 | 0 |
| S. barberi - a | 5 | bell bean | 7 | control | 0 | 0 |
| S. barberi - a | 5 | bell bean | 8 | control | 0 | 0 |
| S. barberi - a | 5 | bell bean | 9 | control | 0 | 0 |
| S. barberi - a | 5 | bell bean | 10 | control | 0 | 0 |
| S. barberi - a | a | bell bean | 1 | test | 0 | 0 |
| S. barberi - a | a | bell bean | 2 | test | 1 | 0 |
| S. barberi - a | a | bell bean | 3 | test | 0 | 1 |
| S. barberi - a | a | bell bean | 4 | test | 0 | 0 |
| S. barberi - a | a | bell bean | 5 | test | 0 | 1 |
| S. barberi - a | a | bell bean | 6 | test | 0 | 0 |
| S. barberi - a | a | bell bean | 7 | test | 1 | 0 |
| S. barberi - a | a | bell bean | 8 | test | 1 | 0 |
| S. barberi - a | a | bell bean | 9 | test | 0 | 0 |
| S. barberi - a | a | bell bean | 10 | test | 0 | 1 |
| S. barberi - a | a | bell bean | 1 | control | 1 | 0 |
| S. barberi - a | a | bell bean | 2 | control | 0 | 0 |
| S. barberi - a | a | bell bean | 3 | control | 0 | 0 |
| S. barberi - a | a | bell bean | 4 | control | 0 | 0 |
| S. barberi - a | a | bell bean | 5 | control | 0 | 0 |
| S. barberi - a | a | bell bean | 6 | control | 1 | 0 |
| S. barberi - a | a | bell bean | 7 | control | 0 | 0 |
| S. barberi - a | a | bell bean | 8 | control | 0 | 0 |
| S. barberi - a | a | bell bean | 9 | control | 1 | 0 |
| S. barberi - a | a | bell bean | 10 | control | 1 | 0 |
| Z. renardii - l | 1 | petri dish | 1 | test | 1 | 0 |
| Z. renardii - l | 1 | petri dish | 2 | test | 1 | 0 |
| Z. renardii - l | 1 | petri dish | 3 | test | 1 | 0 |
| Z. renardii - l | 1 | petri dish | 4 | test | 1 | 0 |
| Z. renardii - l | 1 | petri dish | 5 | test | 1 | 0 |
| Z. renardii - l | 1 | petri dish | 6 | test | 1 | 0 |
| Z. renardii - l | 1 | petri dish | 7 | test | 0 | 1 |
| Z. renardii - l | 1 | petri dish | 8 | test | 1 | 0 |
| Z. renardii - l | 1 | petri dish | 9 | test | 0 | 0 |
| Z. renardii - l | 1 | petri dish | 10 | test | 0 | 0 |
| Z. renardii - l | 1 | petri dish | 1 | control | 0 | 0 |
| Z. renardii - l | 1 | petri dish | 2 | control | 0 | 0 |
| Z. renardii - l | 1 | petri dish | 3 | control | 0 | 0 |
| Z. renardii - l | 1 | petri dish | 4 | control | 0 | 0 |
| Z. renardii - l | 1 | petri dish | 5 | control | 0 | 0 |
| Z. renardii - l | 2 | petri dish | 1 | test | 0 | 1 |
| Z. renardii - l | 2 | petri dish | 2 | test | 1 | 0 |
| Z. renardii - l | 2 | petri dish | 3 | test | 1 | 0 |
| Z. renardii - l | 2 | petri dish | 4 | test | 1 | 0 |
| Z. renardii - l | 2 | petri dish | 5 | test | 0 | 0 |
| Z. renardii - l | 2 | petri dish | 6 | test | 1 | 0 |
| Z. renardii - l | 2 | petri dish | 7 | test | 0 | 0 |
| Z. renardii - l | 2 | petri dish | 8 | test | 1 | 0 |
| Z. renardii - l | 2 | petri dish | 9 | test | 1 | 0 |
| Z. renardii - l | 2 | petri dish | 10 | test | 1 | 0 |
| Z. renardii - l | 2 | petri dish | 1 | control | 0 | 0 |
| Z. renardii - l | 2 | petri dish | 2 | control | 0 | 0 |
| Z. renardii - l | 2 | petri dish | 3 | control | 0 | 0 |
| Z. renardii - l | 2 | petri dish | 4 | control | 0 | 0 |
| Z. renardii - l | 2 | petri dish | 5 | control | 0 | 0 |
| Z. renardii - l | 3 | petri dish | 1 | test | 0 | 0 |
| Z. renardii - l | 3 | petri dish | 2 | test | 0 | 0 |
| Z. renardii - l | 3 | petri dish | 3 | test | 1 | 0 |
| Z. renardii - l | 3 | petri dish | 4 | test | 0 | 0 |
| Z. renardii - l | 3 | petri dish | 5 | test | 0 | 0 |
| Z. renardii - l | 3 | petri dish | 6 | test | 1 | 0 |
| Z. renardii - l | 3 | petri dish | 7 | test | 0 | 0 |
| Z. renardii - l | 3 | petri dish | 8 | test | 0 | 0 |
| Z. renardii - l | 3 | petri dish | 9 | test | 1 | 0 |
| Z. renardii - l | 3 | petri dish | 10 | test | 0 | 0 |
| Z. renardii - l | 3 | petri dish | 1 | control | 0 | 0 |
| Z. renardii - l | 3 | petri dish | 2 | control | 0 | 0 |
| Z. renardii - l | 3 | petri dish | 3 | control | 1 | 0 |
| Z. renardii - l | 3 | petri dish | 4 | control | 0 | 0 |
| Z. renardii - l | 3 | petri dish | 5 | control | 0 | 0 |
| Z. renardii - l | 4 | petri dish | 1 | test | 0 | 0 |
| Z. renardii - l | 4 | petri dish | 2 | test | 0 | 0 |
| Z. renardii - l | 4 | petri dish | 3 | test | 0 | 0 |
| Z. renardii - l | 4 | petri dish | 4 | test | 0 | 1 |
| Z. renardii - l | 4 | petri dish | 5 | test | 0 | 0 |
| Z. renardii - l | 4 | petri dish | 6 | test | 0 | 0 |
| Z. renardii - l | 4 | petri dish | 7 | test | 0 | 0 |
| Z. renardii - l | 4 | petri dish | 8 | test | 0 | 0 |
| Z. renardii - l | 4 | petri dish | 9 | test | 0 | 1 |
| Z. renardii - l | 4 | petri dish | 10 | test | 0 | 0 |
| Z. renardii - l | 4 | petri dish | 1 | control | 0 | 0 |
| Z. renardii - l | 4 | petri dish | 2 | control | 1 | 0 |
| Z. renardii - l | 4 | petri dish | 3 | control | 0 | 0 |
| Z. renardii - l | 4 | petri dish | 4 | control | 0 | 0 |
| Z. renardii - l | 4 | petri dish | 5 | control | 0 | 0 |
| Z. renardii - l | 5 | petri dish | 1 | test | 0 | 0 |
| Z. renardii - l | 5 | petri dish | 2 | test | 0 | 0 |
| Z. renardii - l | 5 | petri dish | 3 | test | 0 | 1 |
| Z. renardii - l | 5 | petri dish | 4 | test | 0 | 0 |
| Z. renardii - l | 5 | petri dish | 5 | test | 0 | 0 |
| Z. renardii - l | 5 | petri dish | 6 | test | 0 | 0 |
| Z. renardii - l | 5 | petri dish | 7 | test | 0 | 0 |
| Z. renardii - l | 5 | petri dish | 8 | test | 0 | 1 |
| Z. renardii - l | 5 | petri dish | 9 | test | 1 | 0 |
| Z. renardii - l | 5 | petri dish | 10 | test | 0 | 0 |
| Z. renardii - l | 5 | petri dish | 1 | control | 0 | 0 |
| Z. renardii - l | 5 | petri dish | 2 | control | 0 | 0 |
| Z. renardii - l | 5 | petri dish | 3 | control | 0 | 0 |
| Z. renardii - l | 5 | petri dish | 4 | control | 0 | 0 |
| Z. renardii - l | 5 | petri dish | 5 | control | 0 | 0 |
| Z. renardii - l | a | petri dish | 1 | test | 1 | 0 |
| Z. renardii - l | a | petri dish | 2 | test | 0 | 0 |
| Z. renardii - l | a | petri dish | 3 | test | 1 | 0 |
| Z. renardii - l | a | petri dish | 4 | test | 0 | 1 |
| Z. renardii - l | a | petri dish | 5 | test | 0 | 1 |
| Z. renardii - l | a | petri dish | 6 | test | 1 | 0 |
| Z. renardii - l | a | petri dish | 7 | test | 0 | 0 |
| Z. renardii - l | a | petri dish | 8 | test | 0 | 0 |
| Z. renardii - l | a | petri dish | 9 | test | 0 | 0 |
| Z. renardii - l | a | petri dish | 10 | test | 0 | 0 |
| Z. renardii - l | a | petri dish | 1 | control | 1 | 0 |
| Z. renardii - l | a | petri dish | 2 | control | 0 | 0 |
| Z. renardii - l | a | petri dish | 3 | control | 0 | 0 |
| Z. renardii - l | a | petri dish | 4 | control | 1 | 0 |
| Z. renardii - l | a | petri dish | 5 | control | 0 | 0 |
| Z. renardii - l | 1 | bell bean | 1 | test | 0 | 0 |
| Z. renardii - l | 1 | bell bean | 2 | test | 0 | 0 |
| Z. renardii - l | 1 | bell bean | 3 | test | 0 | 0 |
| Z. renardii - l | 1 | bell bean | 4 | test | 0 | 0 |
| Z. renardii - l | 1 | bell bean | 5 | test | 1 | 0 |
| Z. renardii - l | 1 | bell bean | 6 | test | 0 | 0 |
| Z. renardii - l | 1 | bell bean | 7 | test | 0 | 0 |
| Z. renardii - l | 1 | bell bean | 8 | test | 0 | 0 |
| Z. renardii - l | 1 | bell bean | 9 | test | 0 | 0 |
| Z. renardii - l | 1 | bell bean | 10 | test | 0 | 0 |
| Z. renardii - l | 1 | bell bean | 1 | control | 0 | 0 |
| Z. renardii - l | 1 | bell bean | 2 | control | 0 | 0 |
| Z. renardii - l | 1 | bell bean | 3 | control | 0 | 0 |
| Z. renardii - l | 1 | bell bean | 4 | control | 0 | 0 |
| Z. renardii - l | 1 | bell bean | 5 | control | 0 | 0 |
| Z. renardii - l | 1 | bell bean | 6 | control | 1 | 0 |
| Z. renardii - l | 1 | bell bean | 7 | control | 0 | 0 |
| Z. renardii - l | 1 | bell bean | 8 | control | 0 | 0 |
| Z. renardii - l | 1 | bell bean | 9 | control | 0 | 0 |
| Z. renardii - l | 1 | bell bean | 10 | control | 0 | 0 |
| Z. renardii - l | 2 | bell bean | 1 | test | 0 | 1 |
| Z. renardii - l | 2 | bell bean | 2 | test | 0 | 0 |
| Z. renardii - l | 2 | bell bean | 3 | test | 0 | 0 |
| Z. renardii - l | 2 | bell bean | 4 | test | 1 | 0 |
| Z. renardii - l | 2 | bell bean | 5 | test | 1 | 0 |
| Z. renardii - l | 2 | bell bean | 6 | test | 0 | 0 |
| Z. renardii - l | 2 | bell bean | 7 | test | 0 | 1 |
| Z. renardii - l | 2 | bell bean | 8 | test | 1 | 0 |
| Z. renardii - l | 2 | bell bean | 9 | test | 0 | 0 |
| Z. renardii - l | 2 | bell bean | 10 | test | 0 | 0 |
| Z. renardii - l | 2 | bell bean | 1 | control | 0 | 0 |
| Z. renardii - l | 2 | bell bean | 2 | control | 0 | 0 |
| Z. renardii - l | 2 | bell bean | 3 | control | 0 | 0 |
| Z. renardii - l | 2 | bell bean | 4 | control | 0 | 0 |
| Z. renardii - l | 2 | bell bean | 5 | control | 0 | 0 |
| Z. renardii - l | 2 | bell bean | 6 | control | 1 | 0 |
| Z. renardii - l | 2 | bell bean | 7 | control | 0 | 0 |
| Z. renardii - l | 2 | bell bean | 8 | control | 0 | 0 |
| Z. renardii - l | 2 | bell bean | 9 | control | 0 | 0 |
| Z. renardii - l | 2 | bell bean | 10 | control | 0 | 0 |
| Z. renardii - l | 3 | bell bean | 1 | test | 0 | 0 |
| Z. renardii - l | 3 | bell bean | 2 | test | 0 | 0 |
| Z. renardii - l | 3 | bell bean | 3 | test | 0 | 0 |
| Z. renardii - l | 3 | bell bean | 4 | test | 1 | 0 |
| Z. renardii - l | 3 | bell bean | 5 | test | 1 | 1 |
| Z. renardii - l | 3 | bell bean | 6 | test | 0 | 0 |
| Z. renardii - l | 3 | bell bean | 7 | test | 1 | 0 |
| Z. renardii - l | 3 | bell bean | 8 | test | 0 | 0 |
| Z. renardii - l | 3 | bell bean | 9 | test | 0 | 0 |
| Z. renardii - l | 3 | bell bean | 10 | test | 0 | 0 |
| Z. renardii - l | 3 | bell bean | 1 | control | 0 | 0 |
| Z. renardii - l | 3 | bell bean | 2 | control | 0 | 0 |
| Z. renardii - l | 3 | bell bean | 3 | control | 0 | 0 |
| Z. renardii - l | 3 | bell bean | 4 | control | 0 | 0 |
| Z. renardii - l | 3 | bell bean | 5 | control | 0 | 0 |
| Z. renardii - l | 3 | bell bean | 6 | control | 0 | 0 |
| Z. renardii - l | 3 | bell bean | 7 | control | 0 | 0 |
| Z. renardii - l | 3 | bell bean | 8 | control | 0 | 0 |
| Z. renardii - l | 3 | bell bean | 9 | control | 0 | 0 |
| Z. renardii - l | 3 | bell bean | 10 | control | 0 | 0 |
| Z. renardii - l | 4 | bell bean | 1 | test | 0 | 0 |
| Z. renardii - l | 4 | bell bean | 2 | test | 0 | 0 |
| Z. renardii - l | 4 | bell bean | 3 | test | 0 | 0 |
| Z. renardii - l | 4 | bell bean | 4 | test | 0 | 0 |
| Z. renardii - l | 4 | bell bean | 5 | test | 0 | 0 |
| Z. renardii - l | 4 | bell bean | 6 | test | 0 | 0 |
| Z. renardii - l | 4 | bell bean | 7 | test | 0 | 0 |
| Z. renardii - l | 4 | bell bean | 8 | test | 0 | 0 |
| Z. renardii - l | 4 | bell bean | 9 | test | 0 | 0 |
| Z. renardii - l | 4 | bell bean | 10 | test | 0 | 0 |
| Z. renardii - l | 4 | bell bean | 1 | control | 1 | 0 |
| Z. renardii - l | 4 | bell bean | 2 | control | 0 | 0 |
| Z. renardii - l | 4 | bell bean | 3 | control | 0 | 0 |
| Z. renardii - l | 4 | bell bean | 4 | control | 0 | 0 |
| Z. renardii - l | 4 | bell bean | 5 | control | 1 | 0 |
| Z. renardii - l | 4 | bell bean | 6 | control | 0 | 0 |
| Z. renardii - l | 4 | bell bean | 7 | control | 0 | 0 |
| Z. renardii - l | 4 | bell bean | 8 | control | 0 | 0 |
| Z. renardii - l | 4 | bell bean | 9 | control | 0 | 0 |
| Z. renardii - l | 4 | bell bean | 10 | control | 0 | 0 |
| Z. renardii - l | 5 | bell bean | 1 | test | 0 | 0 |
| Z. renardii - l | 5 | bell bean | 2 | test | 0 | 0 |
| Z. renardii - l | 5 | bell bean | 3 | test | 0 | 0 |
| Z. renardii - l | 5 | bell bean | 4 | test | 0 | 0 |
| Z. renardii - l | 5 | bell bean | 5 | test | 0 | 0 |
| Z. renardii - l | 5 | bell bean | 6 | test | 0 | 0 |
| Z. renardii - l | 5 | bell bean | 7 | test | 0 | 0 |
| Z. renardii - l | 5 | bell bean | 8 | test | 0 | 0 |
| Z. renardii - l | 5 | bell bean | 9 | test | 0 | 0 |
| Z. renardii - l | 5 | bell bean | 10 | test | 0 | 0 |
| Z. renardii - l | 5 | bell bean | 1 | control | 0 | 0 |
| Z. renardii - l | 5 | bell bean | 2 | control | 0 | 0 |
| Z. renardii - l | 5 | bell bean | 3 | control | 0 | 0 |
| Z. renardii - l | 5 | bell bean | 4 | control | 0 | 0 |
| Z. renardii - l | 5 | bell bean | 5 | control | 1 | 0 |
| Z. renardii - l | 5 | bell bean | 6 | control | 0 | 0 |
| Z. renardii - l | 5 | bell bean | 7 | control | 0 | 0 |
| Z. renardii - l | 5 | bell bean | 8 | control | 0 | 0 |
| Z. renardii - l | 5 | bell bean | 9 | control | 0 | 0 |
| Z. renardii - l | 5 | bell bean | 10 | control | 0 | 0 |
| Z. renardii - l | a | bell bean | 1 | test | 0 | 0 |
| Z. renardii - l | a | bell bean | 2 | test | 0 | 0 |
| Z. renardii - l | a | bell bean | 3 | test | 0 | 1 |
| Z. renardii - l | a | bell bean | 4 | test | 0 | 0 |
| Z. renardii - l | a | bell bean | 5 | test | 0 | 1 |
| Z. renardii - l | a | bell bean | 6 | test | 0 | 0 |
| Z. renardii - l | a | bell bean | 7 | test | 1 | 0 |
| Z. renardii - l | a | bell bean | 8 | test | 0 | 0 |
| Z. renardii - l | a | bell bean | 9 | test | 1 | 0 |
| Z. renardii - l | a | bell bean | 10 | test | 0 | 1 |
| Z. renardii - l | a | bell bean | 1 | control | 0 | 0 |
| Z. renardii - l | a | bell bean | 2 | control | 1 | 0 |
| Z. renardii - l | a | bell bean | 3 | control | 0 | 0 |
| Z. renardii - l | a | bell bean | 4 | control | 0 | 0 |
| Z. renardii - l | a | bell bean | 5 | control | 0 | 0 |
| Z. renardii - l | a | bell bean | 6 | control | 0 | 0 |
| Z. renardii - l | a | bell bean | 7 | control | 0 | 0 |
| Z. renardii - l | a | bell bean | 8 | control | 0 | 0 |
| Z. renardii - l | a | bell bean | 9 | control | 0 | 0 |
| Z. renardii - l | a | bell bean | 10 | control | 0 | 0 |
| C. montrouzieri - l | 1 | petri dish | 1 | test | 0 | 0 |
| C. montrouzieri - l | 1 | petri dish | 2 | test | 0 | 0 |
| C. montrouzieri - l | 1 | petri dish | 3 | test | 0 | 0 |
| C. montrouzieri - l | 1 | petri dish | 4 | test | 0 | 0 |
| C. montrouzieri - l | 1 | petri dish | 5 | test | 0 | 0 |
| C. montrouzieri - l | 1 | petri dish | 6 | test | 0 | 0 |
| C. montrouzieri - l | 1 | petri dish | 7 | test | 0 | 0 |
| C. montrouzieri - l | 1 | petri dish | 8 | test | 0 | 0 |
| C. montrouzieri - l | 1 | petri dish | 9 | test | 0 | 0 |
| C. montrouzieri - l | 1 | petri dish | 10 | test | 0 | 0 |
| C. montrouzieri - l | 1 | petri dish | 1 | control | 0 | 0 |
| C. montrouzieri - l | 1 | petri dish | 2 | control | 0 | 0 |
| C. montrouzieri - l | 1 | petri dish | 3 | control | 0 | 0 |
| C. montrouzieri - l | 1 | petri dish | 4 | control | 0 | 0 |
| C. montrouzieri - l | 1 | petri dish | 5 | control | 0 | 0 |
| C. montrouzieri - l | 2 | petri dish | 1 | test | 0 | 0 |
| C. montrouzieri - l | 2 | petri dish | 2 | test | 1 | 0 |
| C. montrouzieri - l | 2 | petri dish | 3 | test | 0 | 0 |
| C. montrouzieri - l | 2 | petri dish | 4 | test | 0 | 0 |
| C. montrouzieri - l | 2 | petri dish | 5 | test | 0 | 0 |
| C. montrouzieri - l | 2 | petri dish | 6 | test | 0 | 0 |
| C. montrouzieri - l | 2 | petri dish | 7 | test | 0 | 1 |
| C. montrouzieri - l | 2 | petri dish | 8 | test | 0 | 0 |
| C. montrouzieri - l | 2 | petri dish | 9 | test | 0 | 0 |
| C. montrouzieri - l | 2 | petri dish | 10 | test | 1 | 0 |
| C. montrouzieri - l | 2 | petri dish | 1 | control | 0 | 0 |
| C. montrouzieri - l | 2 | petri dish | 2 | control | 0 | 0 |
| C. montrouzieri - l | 2 | petri dish | 3 | control | 0 | 0 |
| C. montrouzieri - l | 2 | petri dish | 4 | control | 0 | 0 |
| C. montrouzieri - l | 2 | petri dish | 5 | control | 0 | 0 |
| C. montrouzieri - l | 3 | petri dish | 1 | test | 0 | 0 |
| C. montrouzieri - l | 3 | petri dish | 2 | test | 0 | 0 |
| C. montrouzieri - l | 3 | petri dish | 3 | test | 0 | 0 |
| C. montrouzieri - l | 3 | petri dish | 4 | test | 0 | 0 |
| C. montrouzieri - l | 3 | petri dish | 5 | test | 0 | 0 |
| C. montrouzieri - l | 3 | petri dish | 6 | test | 0 | 0 |
| C. montrouzieri - l | 3 | petri dish | 7 | test | 0 | 0 |
| C. montrouzieri - l | 3 | petri dish | 8 | test | 0 | 0 |
| C. montrouzieri - l | 3 | petri dish | 9 | test | 0 | 0 |
| C. montrouzieri - l | 3 | petri dish | 10 | test | 0 | 0 |
| C. montrouzieri - l | 3 | petri dish | 1 | control | 0 | 0 |
| C. montrouzieri - l | 3 | petri dish | 2 | control | 0 | 0 |
| C. montrouzieri - l | 3 | petri dish | 3 | control | 0 | 0 |
| C. montrouzieri - l | 3 | petri dish | 4 | control | 0 | 0 |
| C. montrouzieri - l | 3 | petri dish | 5 | control | 0 | 0 |
| C. montrouzieri - l | 4 | petri dish | 1 | test | 0 | 0 |
| C. montrouzieri - l | 4 | petri dish | 2 | test | 0 | 0 |
| C. montrouzieri - l | 4 | petri dish | 3 | test | 0 | 0 |
| C. montrouzieri - l | 4 | petri dish | 4 | test | 0 | 0 |
| C. montrouzieri - l | 4 | petri dish | 5 | test | 0 | 0 |
| C. montrouzieri - l | 4 | petri dish | 6 | test | 0 | 0 |
| C. montrouzieri - l | 4 | petri dish | 7 | test | 0 | 0 |
| C. montrouzieri - l | 4 | petri dish | 8 | test | 0 | 0 |
| C. montrouzieri - l | 4 | petri dish | 9 | test | 1 | 0 |
| C. montrouzieri - l | 4 | petri dish | 10 | test | 1 | 0 |
| C. montrouzieri - l | 4 | petri dish | 1 | control | 0 | 0 |
| C. montrouzieri - l | 4 | petri dish | 2 | control | 0 | 0 |
| C. montrouzieri - l | 4 | petri dish | 3 | control | 0 | 0 |
| C. montrouzieri - l | 4 | petri dish | 4 | control | 0 | 0 |
| C. montrouzieri - l | 4 | petri dish | 5 | control | 0 | 0 |
| C. montrouzieri - l | 5 | petri dish | 1 | test | 0 | 0 |
| C. montrouzieri - l | 5 | petri dish | 2 | test | 0 | 0 |
| C. montrouzieri - l | 5 | petri dish | 3 | test | 0 | 0 |
| C. montrouzieri - l | 5 | petri dish | 4 | test | 1 | 0 |
| C. montrouzieri - l | 5 | petri dish | 5 | test | 0 | 0 |
| C. montrouzieri - l | 5 | petri dish | 6 | test | 1 | 0 |
| C. montrouzieri - l | 5 | petri dish | 7 | test | 0 | 0 |
| C. montrouzieri - l | 5 | petri dish | 8 | test | 0 | 0 |
| C. montrouzieri - l | 5 | petri dish | 9 | test | 0 | 0 |
| C. montrouzieri - l | 5 | petri dish | 10 | test | 0 | 0 |
| C. montrouzieri - l | 5 | petri dish | 1 | control | 0 | 0 |
| C. montrouzieri - l | 5 | petri dish | 2 | control | 0 | 0 |
| C. montrouzieri - l | 5 | petri dish | 3 | control | 0 | 0 |
| C. montrouzieri - l | 5 | petri dish | 4 | control | 0 | 0 |
| C. montrouzieri - l | 5 | petri dish | 5 | control | 0 | 0 |
| C. montrouzieri - l | a | petri dish | 1 | test | 0 | 0 |
| C. montrouzieri - l | a | petri dish | 2 | test | 0 | 0 |
| C. montrouzieri - l | a | petri dish | 3 | test | 0 | 0 |
| C. montrouzieri - l | a | petri dish | 4 | test | 0 | 0 |
| C. montrouzieri - l | a | petri dish | 5 | test | 0 | 0 |
| C. montrouzieri - l | a | petri dish | 6 | test | 0 | 0 |
| C. montrouzieri - l | a | petri dish | 7 | test | 0 | 0 |
| C. montrouzieri - l | a | petri dish | 8 | test | 0 | 0 |
| C. montrouzieri - l | a | petri dish | 9 | test | 0 | 0 |
| C. montrouzieri - l | a | petri dish | 10 | test | 0 | 0 |
| C. montrouzieri - l | a | petri dish | 1 | control | 0 | 0 |
| C. montrouzieri - l | a | petri dish | 2 | control | 0 | 0 |
| C. montrouzieri - l | a | petri dish | 3 | control | 1 | 0 |
| C. montrouzieri - l | a | petri dish | 4 | control | 0 | 0 |
| C. montrouzieri - l | a | petri dish | 5 | control | 0 | 0 |
| C. montrouzieri - l | 1 | bell bean | 1 | test | 1 | 0 |
| C. montrouzieri - l | 1 | bell bean | 2 | test | 0 | 0 |
| C. montrouzieri - l | 1 | bell bean | 3 | test | 0 | 0 |
| C. montrouzieri - l | 1 | bell bean | 4 | test | 0 | 0 |
| C. montrouzieri - l | 1 | bell bean | 5 | test | 0 | 0 |
| C. montrouzieri - l | 1 | bell bean | 6 | test | 0 | 0 |
| C. montrouzieri - l | 1 | bell bean | 7 | test | 0 | 0 |
| C. montrouzieri - l | 1 | bell bean | 8 | test | 0 | 0 |
| C. montrouzieri - l | 1 | bell bean | 9 | test | 0 | 0 |
| C. montrouzieri - l | 1 | bell bean | 10 | test | 0 | 0 |
| C. montrouzieri - l | 1 | bell bean | 1 | control | 0 | 0 |
| C. montrouzieri - l | 1 | bell bean | 2 | control | 0 | 0 |
| C. montrouzieri - l | 1 | bell bean | 3 | control | 0 | 0 |
| C. montrouzieri - l | 1 | bell bean | 4 | control | 0 | 0 |
| C. montrouzieri - l | 1 | bell bean | 5 | control | 0 | 0 |
| C. montrouzieri - l | 1 | bell bean | 6 | control | 0 | 0 |
| C. montrouzieri - l | 1 | bell bean | 7 | control | 0 | 0 |
| C. montrouzieri - l | 1 | bell bean | 8 | control | 0 | 0 |
| C. montrouzieri - l | 1 | bell bean | 9 | control | 0 | 0 |
| C. montrouzieri - l | 1 | bell bean | 10 | control | 1 | 0 |
| C. montrouzieri - l | 2 | bell bean | 1 | test | 0 | 0 |
| C. montrouzieri - l | 2 | bell bean | 2 | test | 0 | 0 |
| C. montrouzieri - l | 2 | bell bean | 3 | test | 0 | 0 |
| C. montrouzieri - l | 2 | bell bean | 4 | test | 0 | 0 |
| C. montrouzieri - l | 2 | bell bean | 5 | test | 0 | 0 |
| C. montrouzieri - l | 2 | bell bean | 6 | test | 0 | 0 |
| C. montrouzieri - l | 2 | bell bean | 7 | test | 0 | 0 |
| C. montrouzieri - l | 2 | bell bean | 8 | test | 0 | 0 |
| C. montrouzieri - l | 2 | bell bean | 9 | test | 0 | 0 |
| C. montrouzieri - l | 2 | bell bean | 10 | test | 0 | 0 |
| C. montrouzieri - l | 2 | bell bean | 1 | control | 0 | 0 |
| C. montrouzieri - l | 2 | bell bean | 2 | control | 0 | 0 |
| C. montrouzieri - l | 2 | bell bean | 3 | control | 0 | 0 |
| C. montrouzieri - l | 2 | bell bean | 4 | control | 0 | 0 |
| C. montrouzieri - l | 2 | bell bean | 5 | control | 0 | 0 |
| C. montrouzieri - l | 2 | bell bean | 6 | control | 0 | 0 |
| C. montrouzieri - l | 2 | bell bean | 7 | control | 0 | 0 |
| C. montrouzieri - l | 2 | bell bean | 8 | control | 0 | 0 |
| C. montrouzieri - l | 2 | bell bean | 9 | control | 0 | 0 |
| C. montrouzieri - l | 2 | bell bean | 10 | control | 0 | 0 |
| C. montrouzieri - l | 3 | bell bean | 1 | test | 0 | 0 |
| C. montrouzieri - l | 3 | bell bean | 2 | test | 0 | 0 |
| C. montrouzieri - l | 3 | bell bean | 3 | test | 0 | 0 |
| C. montrouzieri - l | 3 | bell bean | 4 | test | 0 | 0 |
| C. montrouzieri - l | 3 | bell bean | 5 | test | 0 | 0 |
| C. montrouzieri - l | 3 | bell bean | 6 | test | 0 | 0 |
| C. montrouzieri - l | 3 | bell bean | 7 | test | 1 | 0 |
| C. montrouzieri - l | 3 | bell bean | 8 | test | 0 | 0 |
| C. montrouzieri - l | 3 | bell bean | 9 | test | 0 | 0 |
| C. montrouzieri - l | 3 | bell bean | 10 | test | 0 | 0 |
| C. montrouzieri - l | 3 | bell bean | 1 | control | 0 | 0 |
| C. montrouzieri - l | 3 | bell bean | 2 | control | 0 | 0 |
| C. montrouzieri - l | 3 | bell bean | 3 | control | 0 | 0 |
| C. montrouzieri - l | 3 | bell bean | 4 | control | 0 | 0 |
| C. montrouzieri - l | 3 | bell bean | 5 | control | 0 | 0 |
| C. montrouzieri - l | 3 | bell bean | 6 | control | 0 | 0 |
| C. montrouzieri - l | 3 | bell bean | 7 | control | 1 | 0 |
| C. montrouzieri - l | 3 | bell bean | 8 | control | 0 | 0 |
| C. montrouzieri - l | 3 | bell bean | 9 | control | 0 | 0 |
| C. montrouzieri - l | 3 | bell bean | 10 | control | 0 | 0 |
| C. montrouzieri - l | 4 | bell bean | 1 | test | 0 | 0 |
| C. montrouzieri - l | 4 | bell bean | 2 | test | 0 | 0 |
| C. montrouzieri - l | 4 | bell bean | 3 | test | 0 | 0 |
| C. montrouzieri - l | 4 | bell bean | 4 | test | 0 | 0 |
| C. montrouzieri - l | 4 | bell bean | 5 | test | 0 | 0 |
| C. montrouzieri - l | 4 | bell bean | 6 | test | 0 | 0 |
| C. montrouzieri - l | 4 | bell bean | 7 | test | 1 | 0 |
| C. montrouzieri - l | 4 | bell bean | 8 | test | 0 | 0 |
| C. montrouzieri - l | 4 | bell bean | 9 | test | 0 | 0 |
| C. montrouzieri - l | 4 | bell bean | 10 | test | 1 | 0 |
| C. montrouzieri - l | 4 | bell bean | 1 | control | 0 | 0 |
| C. montrouzieri - l | 4 | bell bean | 2 | control | 1 | 0 |
| C. montrouzieri - l | 4 | bell bean | 3 | control | 0 | 0 |
| C. montrouzieri - l | 4 | bell bean | 4 | control | 1 | 0 |
| C. montrouzieri - l | 4 | bell bean | 5 | control | 1 | 0 |
| C. montrouzieri - l | 4 | bell bean | 6 | control | 0 | 0 |
| C. montrouzieri - l | 4 | bell bean | 7 | control | 0 | 0 |
| C. montrouzieri - l | 4 | bell bean | 8 | control | 0 | 0 |
| C. montrouzieri - l | 4 | bell bean | 9 | control | 0 | 0 |
| C. montrouzieri - l | 4 | bell bean | 10 | control | 0 | 0 |
| C. montrouzieri - l | 5 | bell bean | 1 | test | 1 | 0 |
| C. montrouzieri - l | 5 | bell bean | 2 | test | 0 | 0 |
| C. montrouzieri - l | 5 | bell bean | 3 | test | 0 | 0 |
| C. montrouzieri - l | 5 | bell bean | 4 | test | 0 | 0 |
| C. montrouzieri - l | 5 | bell bean | 5 | test | 0 | 0 |
| C. montrouzieri - l | 5 | bell bean | 6 | test | 0 | 0 |
| C. montrouzieri - l | 5 | bell bean | 7 | test | 0 | 0 |
| C. montrouzieri - l | 5 | bell bean | 8 | test | 0 | 0 |
| C. montrouzieri - l | 5 | bell bean | 9 | test | 0 | 0 |
| C. montrouzieri - l | 5 | bell bean | 10 | test | 0 | 0 |
| C. montrouzieri - l | 5 | bell bean | 1 | control | 0 | 0 |
| C. montrouzieri - l | 5 | bell bean | 2 | control | 0 | 0 |
| C. montrouzieri - l | 5 | bell bean | 3 | control | 0 | 0 |
| C. montrouzieri - l | 5 | bell bean | 4 | control | 0 | 0 |
| C. montrouzieri - l | 5 | bell bean | 5 | control | 0 | 0 |
| C. montrouzieri - l | 5 | bell bean | 6 | control | 0 | 0 |
| C. montrouzieri - l | 5 | bell bean | 7 | control | 0 | 0 |
| C. montrouzieri - l | 5 | bell bean | 8 | control | 0 | 0 |
| C. montrouzieri - l | 5 | bell bean | 9 | control | 0 | 0 |
| C. montrouzieri - l | 5 | bell bean | 10 | control | 1 | 0 |
| C. montrouzieri - l | a | bell bean | 1 | test | 0 | 0 |
| C. montrouzieri - l | a | bell bean | 2 | test | 1 | 0 |
| C. montrouzieri - l | a | bell bean | 3 | test | 0 | 0 |
| C. montrouzieri - l | a | bell bean | 4 | test | 0 | 0 |
| C. montrouzieri - l | a | bell bean | 5 | test | 0 | 0 |
| C. montrouzieri - l | a | bell bean | 6 | test | 0 | 0 |
| C. montrouzieri - l | a | bell bean | 7 | test | 0 | 0 |
| C. montrouzieri - l | a | bell bean | 8 | test | 0 | 0 |
| C. montrouzieri - l | a | bell bean | 9 | test | 0 | 0 |
| C. montrouzieri - l | a | bell bean | 10 | test | 0 | 0 |
| C. montrouzieri - l | a | bell bean | 1 | control | 0 | 0 |
| C. montrouzieri - l | a | bell bean | 2 | control | 1 | 0 |
| C. montrouzieri - l | a | bell bean | 3 | control | 0 | 0 |
| C. montrouzieri - l | a | bell bean | 4 | control | 0 | 0 |
| C. montrouzieri - l | a | bell bean | 5 | control | 1 | 0 |
| C. montrouzieri - l | a | bell bean | 6 | control | 0 | 0 |
| C. montrouzieri - l | a | bell bean | 7 | control | 0 | 0 |
| C. montrouzieri - l | a | bell bean | 8 | control | 0 | 0 |
| C. montrouzieri - l | a | bell bean | 9 | control | 0 | 0 |
| C. montrouzieri - l | a | bell bean | 10 | control | 0 | 0 |
